# Optimizing an ethanol-based fixative for enhanced nucleic acid preservation in cervical samples using a central composite design approach

**DOI:** 10.1371/journal.pone.0349088

**Published:** 2026-06-26

**Authors:** Ghazal Sedaghat Shayegan, Pouya Salehipour, Ali Najafi, Mohammad Hossein Modarressi

**Affiliations:** 1 Department of Medical Genetics, School of Medicine, Tehran University of Medical Sciences, Tehran, Iran; 2 Genomics Department, Nilou Medical Laboratory, Tehran, Iran; Dr. Dayaram Patel Pharmacy College, INDIA

## Abstract

**Background:**

To accurately diagnose cervical cancer, high-quality genetic material (DNA and RNA) from clinical samples is crucial. Current preservation methods often have limitations, including poor RNA stability and safety concerns. This study aimed to develop an optimized ethanol-based fixative to preserve DNA and RNA in cervical samples under ambient conditions.

**Methods:**

HeLa cells were fixed in ethanol-based fixatives containing polyhydric compounds (Sorbitol and polyethylene glycol [PEG]) and chelating agents. We used a central composite design (CCD) approach to evaluate the effects of pH, Sorbitol concentration, and PEG concentration on nucleic acid preservation. DNA and RNA quality were assessed using agarose gel electrophoresis, PCR, and real-time PCR. Cellular morphology was evaluated using Papanicolaou-stained slides. HPV genotyping of clinical samples was conducted using real-time PCR.

**Results:**

The optimized fixative, developed in this study consisted of 40% ethanol, 4.3% Sorbitol, 1.2% PEG 8000, with a pH of 5.6. This new formula significantly improved DNA and RNA preservation compared to the commercial solution, PreservCyt. DNA showed high integrity and was successfully amplified in PCR test targeting HPV-18 oncogenes. RNA quality was confirmed through clear 28S and 18S rRNA bands and lower threshold cycle (Ct) values in qRT-PCR. HPV genotyping and morphological analysis revealed excellent preservation, enabling both molecular and cytological evaluations.

**Conclusion:**

The new ethanol-based fixative represents a promising cost-effective and environmentally friendly solution for preserving nucleic acids and cellular morphology in cervical samples. Its good performance under ambient conditions suggests it may serve as an option for Human Papillomavirus (HPV) testing and cervical cancer prevention, particularly in places with limited resources.

## 1. Introduction

Cervical cancer is an important global health issue that particularly affects the female reproductive system. Cervical cancer is the fourth most common cancer in women worldwide, according to the World Health Organization (WHO) scale [1]. Human papillomavirus (HPV) is the primary cause of cervical cancer, which plays a crucial role in its onset. The HPV family currently has 15 HPV genotypes classified as high risk. Among these HPV-16 and HPV-18, contribute to most of the cervical cancer cases. These high-risk HPV variants could cause enduring infections that may ultimately progress to a cancerous state [2].

Advances in molecular diagnostics have facilitated our ability to detect cancer early and assess risk more accurately. We can better understand how cancer develops and predict its progression by identifying the increased expression of *E6* and *E7* oncogenes [3]. Moreover, using RNA-based screening for the human papillomavirus (HPV) shows great potential in predicting when mild lesions might become more severe, helping us to anticipate and manage cancer more effectively [4,5]. However, the accuracy of these diagnostic tests depends on the quality and integrity of the nucleic acids in clinical samples. The degradation of DNA and RNA during sample collection, storage, and processing can affect the accuracy of molecular testing. Therefore, preserving genetic material in its intact form is a critical requirement for effective diagnostic outcomes.

Several molecular methods are approved by the FDA for diagnosing HPV, including nucleic acid hybridization assays, signal amplification assays, and nucleic acid amplification assays. These procedures require that RNA and DNA be kept in an acceptable level of integrity for test [6]. One of the most promising advantages of RNA-based HPV screening is that it can predict when minor cellular abnormalities might turn into serious conditions like cancer. These novel insights could fundamentally change how we prevent HPV from progressing to cancer [3].

Morphological and DNA screenings alone are not sufficient to differentiate high-grade lesions that are likely to persist or progress to invasive cancers. The preservation of HPV mRNA assists in understanding the extent of viral gene expression, which is an essential aspect for early detection and prevention [7]. Despite improvements in sample storage media and preservation methods, challenges still exist [8,9]. Issues such as implementing toxic materials like methanol and maintaining the integrity of genetic information during storage must be addressed in order to ensure the accuracy of cervical cancer tests because DNA and RNA can fragment or alter in a way to produce inaccurate test results.

It is essential that liquid-based solutions for testing be safe for both technicians and the environment. Some solutions, like the methanol-based PreservCyt, despite the handling hazards of methanol, provide satisfactory storage for immersed cytological cervical samples [10]. Current methanol-based preservation methods [11,12], like CytoRich Red and PreservCyt often face handling challenges including safety concerns and environmental impact. Ethanol-based fixatives offer a safer alternative, but some of them might not effectively preserve RNA [13], so their formulation requires optimization to ensure long-term nucleic acid stability under liquid immersion storage at room temperature [14].

We investigated the use of biodegradable and safer substitutes, such as mono and poly alcohols, to develop a suitable preservative in order to address the aforementioned concerns. To further highlight the originality of our strategy, we also included Sorbitol, a polyol and sugar alcohol known for its capacity to preserve cytosolic materials apart from DNA [15]. In this study, we developed a proper preservative for liquid-based Pap test, using a central composite design to systematically evaluate the effects of pH, PEG, and Sorbitol concentrations on sample integrity. Additionally, the optimized formulation was compared with a commercial standard to assess its performance under ambient storage conditions.

## 2. Materials and methods

### 2.1 Sample preparation

All cell culture procedures and clinical sample handling had the ethical approval of the Tehran University of Medical Sciences’ Ethics Committee (IR.TUMS.MEDICINE.REC.1400.118). Studies on human donor cervical samples were performed in accordance with the central principles of the Declaration of Helsinki. For this study 14 cervical exfoliated cells were collected from consenting participants between October 5, 2025, and November 20, 2025, by Nilou Laboratory. All participants have provided written informed consent to the Nilou Laboratory. Samples were collected using a brush and preserved in the novel fixative at room temperature for seven days before being processed. Seven samples were obtained from women presenting symptoms or with previous HPV-positive results, while the remaining seven were from women without known risk factors. Three of these samples, were divided into two portions to allow pairwise comparison between the optimized ethanol-based fixative and the commercial PreservCyt solution.

HeLa human cervical cancer cells (ATCC CCL-2) were obtained from the National Center for Genetic and Biological Resources of Iran. The genome of HeLa cells contains integrated HPV18 DNA, and expresses E6 and E7 oncoproteins. The HeLa cells were cultured in Dulbecco’s Modified Eagle’s Medium (DMEM, Gibco) supplemented with 10% heat-inactivated fetal bovine serum (Biowest) and 10 μL/mL penicillin-streptomycin (Life Technologies, Gibco). After harvest, approximately 5*10^6^ cells were washed with phosphate-buffered saline (PBS), and were re-suspended in 5 mL of ethanol-based fixative containing chelating agents for liquid-based cytology (LBC). Samples were stored at room temperature for one week before further analysis.

### 2.2 DNA and RNA extraction

Fixed cells were centrifuged at 200g for 5 minutes and re-suspended in 100 μL of PBS buffer for further procedures. For genomic DNA isolation using the SinaPure DNA kit (SinaClon EX6001), 25 μL of Proteinase K (SinaClon) was added in the initial steps according to the manufacturer’s procedure. After following the instructions, DNA was dissolved in 100 μL of the elution buffer, then it was treated with 2 μL of RNase A (SinaClon, 20 mg/mL) to remove RNA contamination.

Total RNA was extracted using Trizol (Invitrogen) and diluted in 100 μL of nuclease-free water. Then, it was treated with 2 units of DNase I (SinaClon) in the DNase I reaction buffer. After enzymatic digestion, isopropanol and 70% ethanol were used to concentrate RNA and DNA to remove unwanted substances (enzymes, salts, etc.). The final DNA and RNA were dissolved in 50 μL of nuclease-free water. The isolated RNA was stored at −80°C for downstream analyses.

### 2.3 Evaluating nucleic acid quantity and quality

DNA and RNA concentrations were measured using a NanoDrop™ 2000 spectrophotometer (Thermo Scientific). DNA integrity was evaluated using the system of scaling based on quality. As described previously [16] the intensity of the nucleic acid band after running on agarose gel can be scaled from faint to clear and bright bands. Briefly, 5 μL of a diluted DNA mixture (1 μL of extracted DNA, 3 μL of nuclease-free water, and 1 μL of loading dye) was loaded onto a 1% agarose gel stained with DNA-safe stain. Band intensity was scored semi-quantitatively as 0 (poor), 1 (moderate), or 2 (good) based on the preservation of the DNA. Band-intensity scoring was performed by two independent observers blinded to sample identity.

The presence of distinct RNA bands on agarose gel was used as an indicator of RNA integrity. RNA quality was evaluated by visual inspection of the 28S and 18S rRNA bands following electrophoresis, as reported previously [17].

### 2.4 Fixative screening and selection (Initial screening and selection of fixatives)

To identify an optimal fixative, basal fixatives were initially screened. These contained a range of ethanol concentrations (25%, 30%, 40%, and 50%) and were prepared in 10 mM divalent cation-free phosphate buffer with 2 mM Na2EDTA as a chelating agent. In addition, the effects of different chelating agents (disodium EDTA vs. sodium citrate) on DNA preservation were evaluated. The fixative composition demonstrating the highest efficacy was selected for further optimization using a central composite design (CCD).

### 2.5 Optimization experimental design

Optimization was performed using a central composite design (CCD) implemented in Design-Expert software version 13 with an alpha (α) value of 1.5. Three variables were examined: pH (acidic or neutral), Sorbitol concentration (0.9–4.5%), and PEG 8000 concentration (0.9–4.5%). The acidic pH was maintained using 10 mM acetate buffer, while the neutral pH was achieved with 10 mM phosphate buffer.

A total of 48 experimental runs were conducted using two numeric factors, the effects of PEG 8000 and Sorbitol concentrations, and pH as a categorical factor. The design included 16 center point replicates (8 for each of two pH levels), 12 axial points (6 with high PEG and 6 with high Sorbitol, each with the other factor held at its center level), and 8 factorial runs (Table 1). DNA band intensity and absorbance at 260 nm were used sequentially as score and concentration responses. These data were statistically analyzed with no transformation (S1 Table). Additionally, in twelve experimental runs, one factor (either PEG or Sorbitol) was completely omitted (0%) while the other was maintained at its center level. These supplemental runs were useful to evaluate the baseline response in the absence of one additive. These special runs were compared with the two basal combinations that did not include any additives at two PH levels. This comparison provides more insight into the impact of each component individually and they were analyzed separately to preserve the continuous nature of the CCD (S2 Table).

**Table 1:** CCD experimental runs.

Run Type	Count	Purpose
**Center points** (PEG and Sorbitol middle, 2 pH levels)	16	Estimate pure error, test for curvature, and assess pH-level effects
**Axial runs** (PEG high, Sorbitol middle)	6	Extend PEG range to detect optimal or threshold effects
**Axial runs** (PEG middle, Sorbitol high)	6	Extend Sorbitol range to detect optimal or threshold effects
**Exploratory zero runs** (PEG or Sorbitol = 0%, other at middle level)	12	Investigate baseline effect of each additive individually
**Factorial runs** (high/low combinations)	8	Core CCD factorial structure to model main effects and interactions

### 2.6 Predictive model development and validation

The CCD analysis was carried out based on the factors that were hypothesized to influence DNA preservation: pH, Sorbitol concentration, and PEG 8000 concentration. After completing 48 experiments, a predictive model was generated by omitting 12 exploratory runs, in which one factor (either PEG or Sorbitol) was set to 0% while the other was maintained at its center level. No transition point was chosen to fit the regression model, and then the model was refined by eliminating non-significant terms. The fit statistics for quadratic model is reported in Table 2. This model was then used to establish three optimization targets: (1) minimum PEG with minimum Sorbitol, while the response variables were set to their maximum values (2) minimum PEG with maximum Sorbitol, while achieving maximum response and (3) all factors were set in range, with the goal of maximizing the response (Table 3). Three suggested solutions were validated through assessments of DNA and RNA integrity on agarose gel electrophoresis, concentration measurements by Nano drop, and PCR amplification. The related data are presented in the main text figures. Raw images are available as individual files (S1–S6 Raw Figs) and as a compiled file (S1 File).

**Table 2:** Fit statistics for quadratic model.

Response 1: Score		R²	0.9918	Response 2: Concentration		R²	0.9412
**Mean**	1.28	**Adjusted R²**	0.9893	**Mean**	140.86	**Adjusted R²**	0.9232
**C.V. %**	7.06	**Predicted R²**	0.9786	**C.V. %**	9.76	**Predicted R²**	0.8860
**Std. Dev.**	0.0902	**Adeq Precision**	44.7451	**Std. Dev.**	13.74	**Adeq Precision**	26.9211

**Table 3:** The desirability function: weights/goals for each response.

Solution 1	Goal	Solution 2	Goal	Name	Goal	Lower Limit	Upper Limit	Lower Weight	Upper Weight	Importance
A:PEG8000	minimize	A:PEG8000	minimize	A:PEG8000	in range	0	5.4	1	1	3
B:Sorbitol	minimize	B:Sorbitol	maximize	B:Sorbitol	in range	0	5.4	1	1	3
C:PH	in range	C:PH	in range	C:PH	in range	5.6	7.0	1	1	3
Score	maximize	Score	maximize	Score	maximize	0	2	1	1	5
Concentration	maximize	Concentration	maximize	Concentration	maximize	25	231	1	1	5

### 2.7 PCR analysis

DNA quality was validated through PCR amplification of target genes. Three sets of primers were used: one specific to the HPV-18 *E6* and *E7* oncogenes, which amplified a 906 bp fragment, and two additional sets employed for integrity analysis, amplifying fragments of 1844 bp and 1622 bp, respectively (Table 4). These primers specific for *BCL6* and *SYCP3* were also amplified under the same conditions as those used for the *E6* and *E7* oncogenes. Each 25 μL reaction contained 0.5 μL of template DNA, 0.12 μM of each primer, 12.5 μL Taq DNA Polymerase 2x Master Mix (Ampliqon), and PCR grade water. Cycling conditions included an initial denaturation at 95°C for 5 minutes, followed by 35 cycles of 30 seconds at 95°C, 60 seconds at 58°C, and 30 seconds at 72°C, with a final extension at 72°C for 10 minutes. PCR products were visualized on a 2% agarose gel stained with DNA-safe dye.

**Table 4:** Designed primers and their target locations and PCR product sizes.

Genes	Forward primer	Reverse primer	PCR Product size
**HPV18** **E6-E7**	GTAACCGAAAACGGTCGGGA NC_001357.1: 39...58(20nt)	CCTCCCCGTCTGTACCTTCT NC_001357.1: 944...925(20nt)	906 bp
**HPV18** **E6**	CAGAAACCGTTGAATCCAGCA NC_001357.1: 429...449(21nt)	TTGGAGTCGTTCCTGTCGTG NC_001357.1: 557...538(20nt)	129 bp
**Hpv18** **E7**	GTCACGAGCAATTAAGCGACT NC_001357.1: 669...689(21nt)	GGAATGCTCGAAGGTCGTCT NC_001357.1: 848...829(20nt)	180 bp
**BCL6**	TGGAGCATGTTGTGGACACT NC_000003.12: 187731751...187731732(20nt)	TTGTTCTCCACCACCTCACG NC_000003.12: 187729908...187729927(20nt)	1844 bp
**SYCP3**	GACTGGCTTTTCTCCTGTGC NC_000012.12: 101739499...101739480(20nt)	GATCTTCCACAGACGGCTTC NC_000012.12: 101737878...101737897(20nt)	1622 bp

### 2.8 HPV m-RNA detection on fixed HeLa cells

Quantitative real-time PCR (qRT-PCR) was performed using primers targeting HPV-18 *E6* and *E7*. RNA was treated with DNase to remove genomic DNA contamination, followed by reverse transcription using the TAKARA Reverse Transcription kit. cDNA synthesis employed mixed random and oligo-dT primers. qRT-PCR reactions were conducted using a Rotor-Gene 6000 (Corbett Life Science) under the following conditions: initial denaturation at 95°C for 15 minutes, followed by 40 cycles of 30 seconds at 95°C, 60 seconds at 57°C, and 30 seconds at 72°C.

### 2.9 Morphological preservation analysis

Fixed cervical exfoliated cells were collected from patients in either the optimized ethanol-based fixative or the commercial PreservCyt solution and were analyzed after seven days of storage at room temperature. Slides were prepared using the ThinPrep 2000 Processor (Hologic) and stained with Papanicolaou (Pap) stain. Two independent cytopathologists, blinded to fixative type, evaluated all slides. Each case was presented as two anonymized slides labeled only with letters; raters had no information regarding fixative type. Slides were examined using a 10 × objective magnification for general scanning and 40 × objective magnification for detailed cytomorphological assessment. For each slide, five non-overlapping random fields were evaluated at 40 × .

Assessment focused on five key parameters:

1. Cell Type Representation – adequacy and preservation of superficial, intermediate, and parabasal epithelial cells.
2. Staining Clarity (Nuclear/Cytoplasmic) – intensity and fidelity of nuclear and cytoplasmic staining.
3. Cell Distribution – uniformity of monolayer spread and degree of overlapping or aggregation.
4. Background Cleanliness – presence of obscuring debris, inflammatory material, mucus, or blood.
5. Morphologic Preservation – cell border integrity, cytoplasmic preservation, and absence of degenerative changes.

Five key morphological parameters were scored in Ordinal Scoring Rubric (0–3 Scale). Scores ranged from 0 (poor) to 3 (optimal) for each feature.

The mean scores per morphological feature were compared between raters. Inter-rater agreement was quantified using this formula:


$$\begin{array}{c} Agreement(\%)=\text{1}\text{0}\text{0}\times \left(\text{1}-\frac{\left(|R\text{1}-R\text{2}|\right)}{\text{3}}\right) \end{array}$$

R₁ and R₂ are the mean scores of the two raters for a given feature per slide. The denominator “3” represents the maximum possible difference between two raters.

### 2.10 HPV molecular testing on cervical samples

The Rastin HPV Genotyping Real-Time PCR Kit was used to detect 33 HPV genotypes, including 14 high-risk, 5 intermediate-risk, and 14 low-risk types. DNA was extracted from samples and amplified using the Rotor-Gene Q real-time PCR system according to the manufacturer’s instructions. The kit includes specific primers and probes to detect E6 and E7 genes of 33 HPV types in a multiplex system composed of 8 reaction tubes and 4 fluorescence channels. If the internal control gene, human hemoglobin subunit beta (HBB), is not detected, the sampling process should be questioned because of the possibility of insufficient epithelial cells or high mucus content in the sample.

Amplification cycles included initial Enzyme activation at 95°C for 10 minutes, followed by two steps of amplification, an initial 5 cycles to reduce off-target artifacts, and subsequent 40 cycles of denaturation, annealing, and extension with fluorescence detection. The assay’s limit of detection was 40 copies/mL, ensuring enough analytical sensitivity.

### 2.11 Statistical analysis

Continuous variables were analyzed using Design Expert software after excluding zero-value conditions. Statistical significance was determined using one-way analysis of variance (ANOVA). A p-value < 0.05 was considered statistically significant. Descriptive statistics, including mean, standard deviation (SD), coefficient of variation (CV%), and inter-rater agreement (%), were calculated using Excel.

### 2.12 Ethical approval

This study was approved by the Ethics Committee for Clinical Research at Tehran University of Medical Sciences (IR.TUMS.MEDICINE.REC.1400.118). All procedures involving human participants were conducted in accordance with the principles of the Declaration of Helsinki. Cervical samples were collected at Nilou Laboratory as part of routine clinical procedures. All participants provided written informed consent for both sample collection and their use in research prior to inclusion in the study. To ensure confidentiality, all samples and data were anonymized before being made available to the research team and no personal identifiers were accessible at any stage of the study.

## 3. Results

### 3.1 Evaluation of ethanol concentration and chelating agents

**Fig 1:**
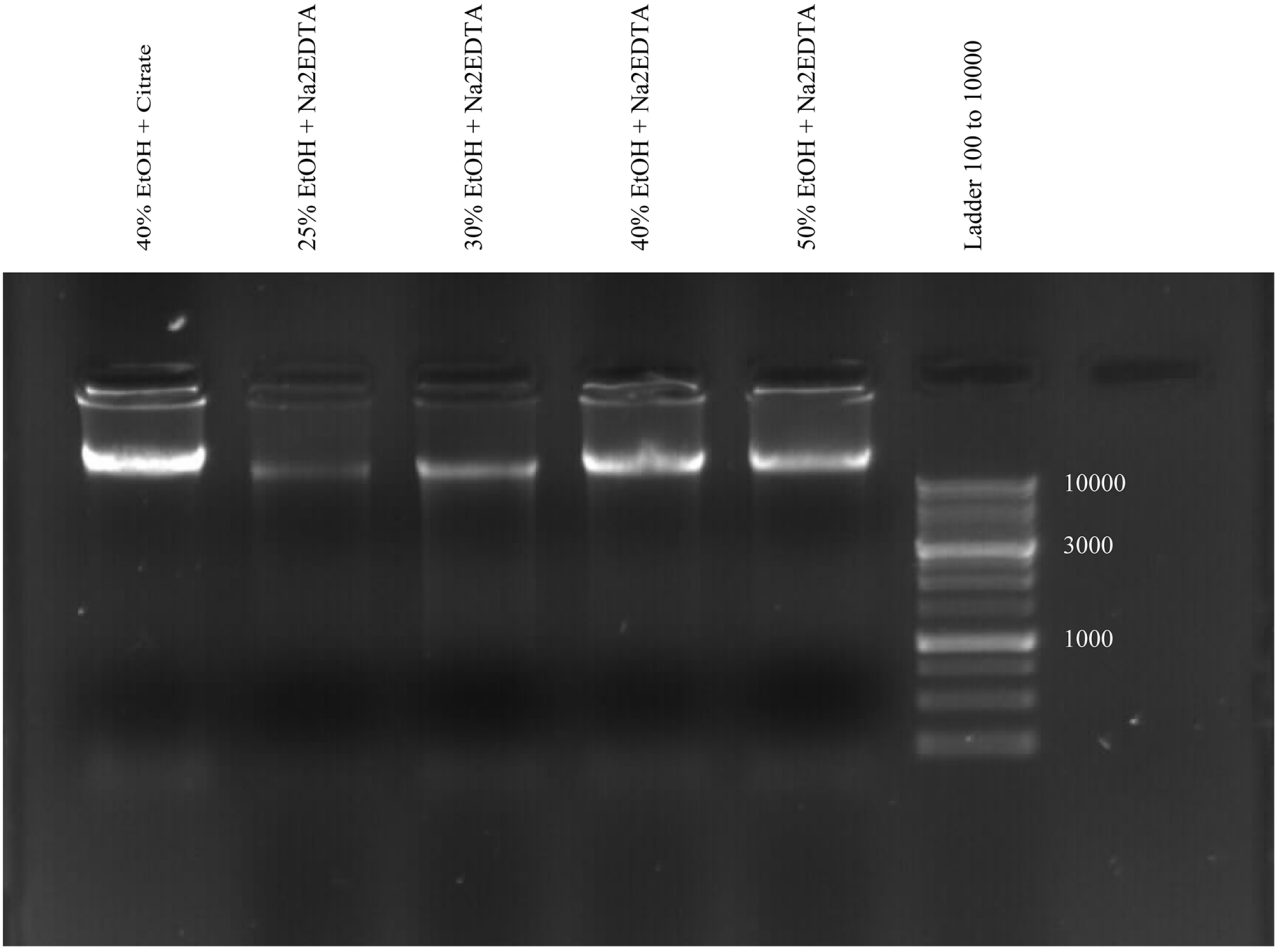
Comparing various ethanol concentration in terms of integrity score. Genomic DNA bands extracted from stored Hela cells in combinations containing: 1- 40% Ethanol in phosphate buffer with 5 mM Citric acid, Score: + 2. 2- 25% Ethanol in phosphate buffer with 2 mM Na2EDTA, Score: 0. 3- 30% Ethanol in phosphate buffer with 2 mM Na2EDTA, Score: + 1. 4- 40% Ethanol in phosphate buffer with 2 mM Na2EDTA, Score: + 2. 5) 50% Ethanol in phosphate buffer with 2 mM Na2EDTA, Score: + 2.

**Fig 2:**
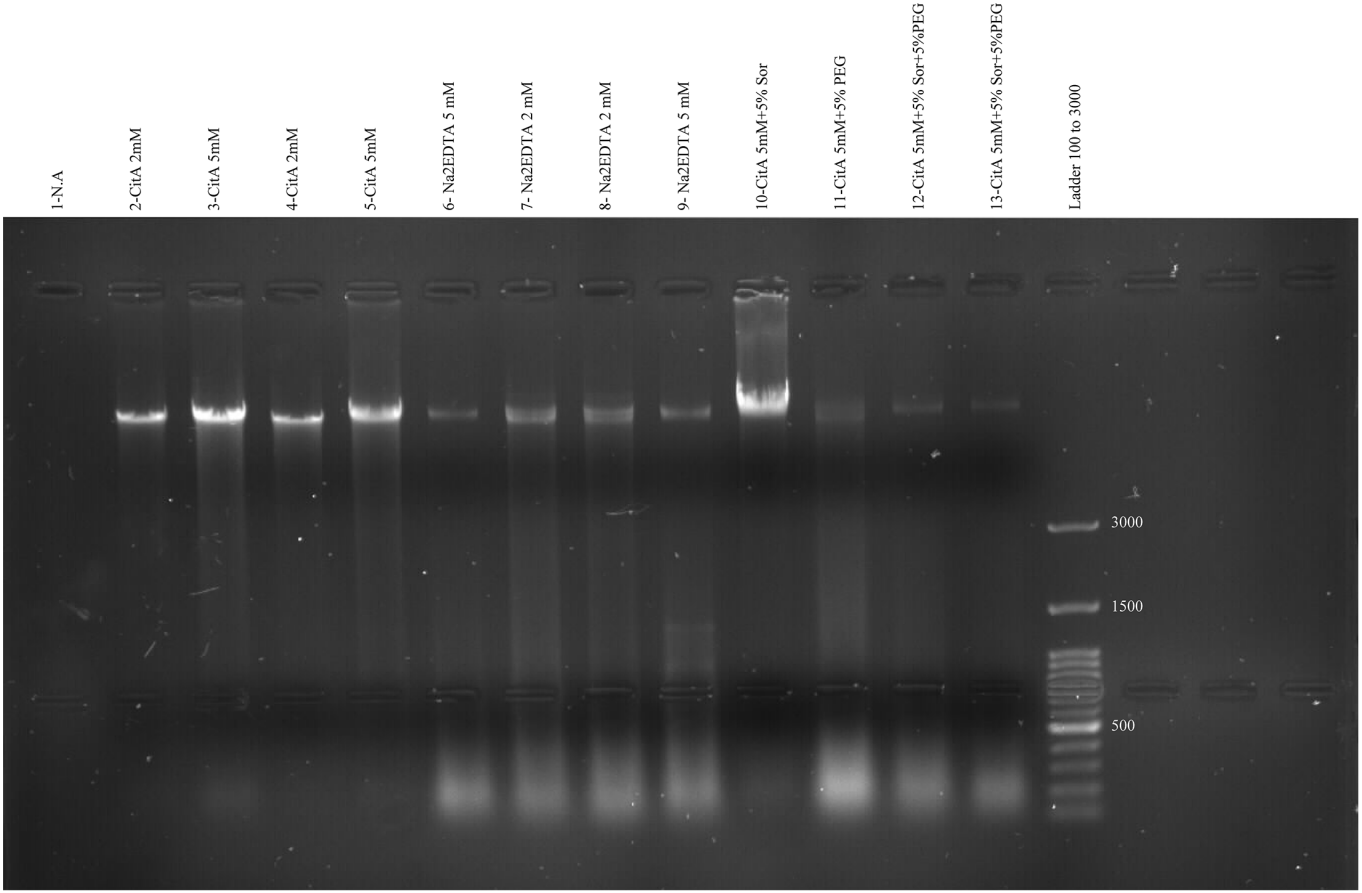
Extracted genomic DNA of fixed HeLa cells in the listed combinations. 1- Empty (Not available N.A.) 2- citric acid 2 mM, Score: + 1, 3- citric acid 5 mM, Score: + 2, 4- replicate of citric acid 2 mM, Score: + 1, 5- replicate of citric acid 5 mM, Score: + 2, 6- Na2EDTA 5mM, Score: 0, 7- Na2EDTA 2mM, Score: + 1, 8- replicate of Na2EDTA 2mM, Score: + 1, 9- replicate of Na2EDTA 5mM, Score: 0, 10- citric acid 5 mM with 5% Sorbitol, Score: + 2, 11-citric acid 5 mM with 5% PEG, Score: 0, 12- citric acid 5 mM with 5% PEG and 5% Sorbitol, Score: 0, 13- replicate of citric acid 5 mM with 5% PEG and 5% Sorbitol, Score: 0.

In the Initial screening process, it was revealed that ethanol concentrations below 30% were ineffective in preserving genomic DNA. It can be inferred by faint DNA bands on agarose gel. In contrast, 40% and 50% ethanol solutions demonstrated higher DNA preservation with intense DNA bands. The concentration of 40% ethanol was chosen because a lower alcohol concentration in the solution reduces the chance of cell shrinkage and flammability risk (Fig 1). Subsequently chelating agents were compared revealing that the concentration of 2mM citric acid performed better than 2mM of Na₂EDTA in protecting DNA. Increasing the concentration of citric acid to 5 mM provided better protection, indicated by higher band intensity and better DNA yield. In contrast, increasing Na2EDTA concentration to 5 mM in the basal solution of 40% ethanol and acetic acid buffer did not resulted in improved preservation (Fig 2).

### 3.2 Impact of formulation variables on DNA preservation: A central composite design approach

**Fig 3:**
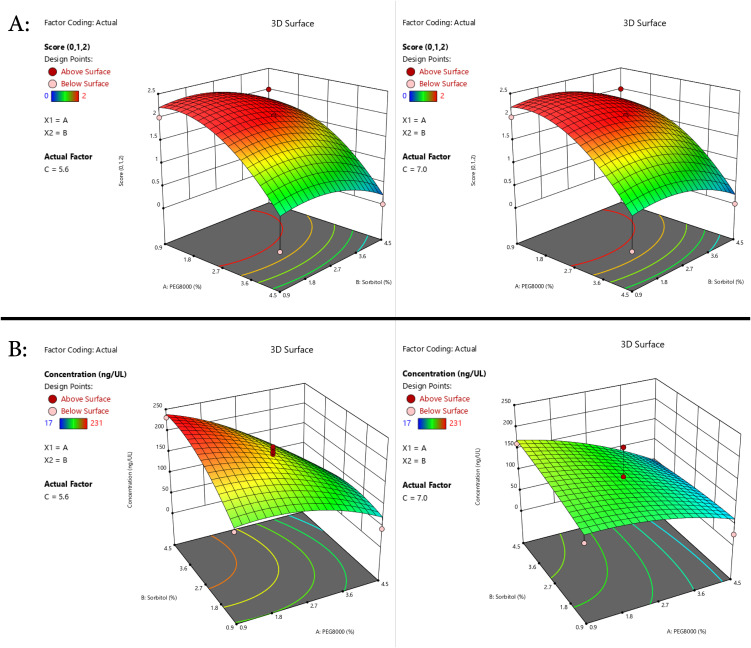
Response surface plot. Represented 3D graphs indicate how PEG8000 and Sorbitol interact and affect each response at two pH levels: acidic and neutral. Regardless of pH, the results for scoring response (A) are comparable, indicating that employing less PEG and more Sorbitol is advantageous. For concentration response (B), acidic pH makes a noticeable difference, with higher Sorbitol and lower PEG concentrations resulting in better performance.

Based on the response 3D surface plot, after input data analysis it seems that PEG and Sorbitol concentration have significant impacts on the responses (Fig 3). The significance of each factor and interaction was evaluated through ANOVA (analysis of variance). Sorbitol concentrations between 1% and 4% and PEG 8000 concentrations below 2% appeared to have a positive effect on the response. Supplementary data from the baseline response analysis (S2 Table) implies that these two variables improved the level of DNA preservation; however, Sorbitol inclusion seems to be more beneficial. Moreover, the interaction between Sorbitol and PEG showed a synergistic effect, enhancing DNA preservation when combined at specific ratios in acidic PH.

### 3.3 Optimization of fixative combinations using ANOVA based on response goals

**Fig 4:**
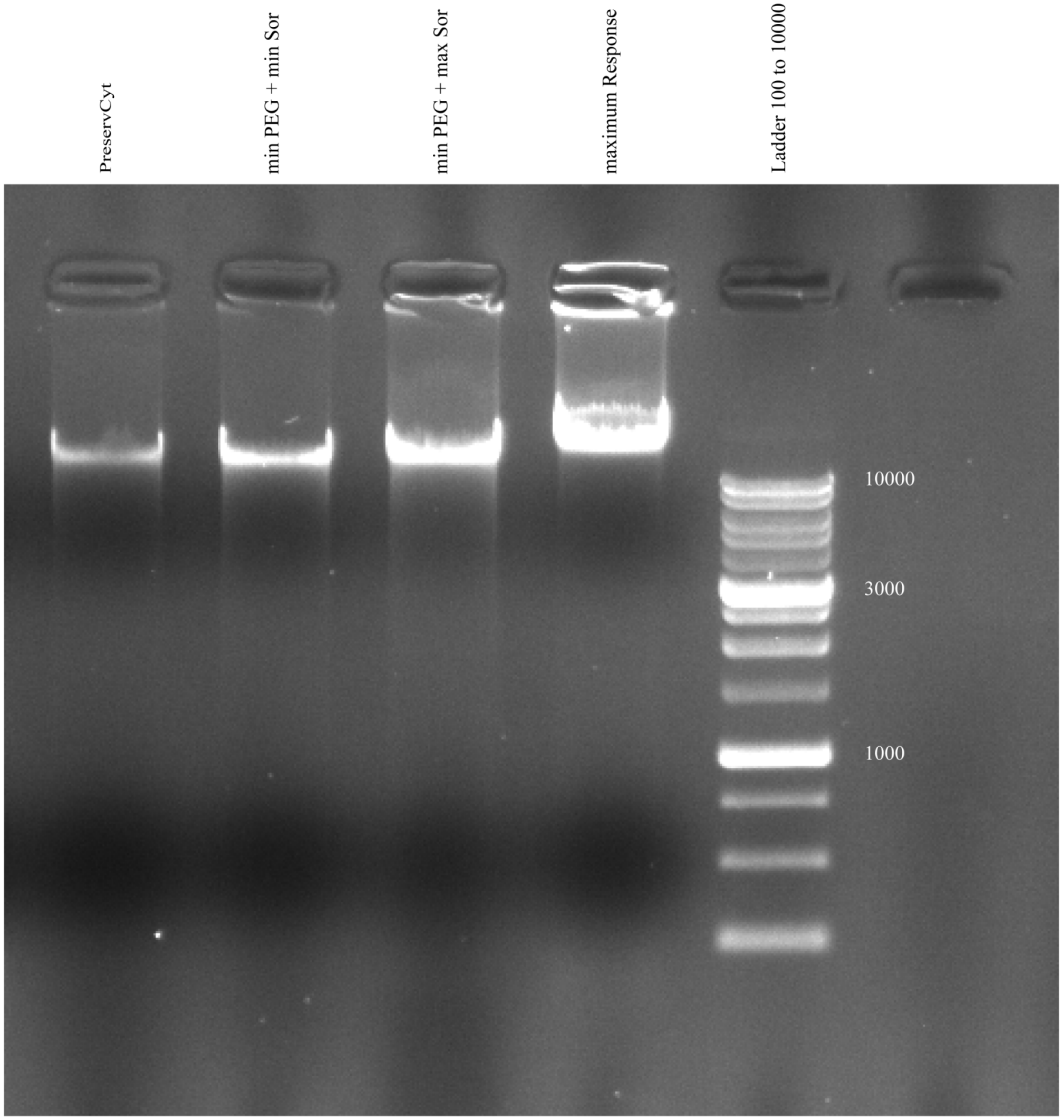
Evaluating optimization targets: Final solutions vs. standard. This Figure demonstrates bands of genomic DNA with different intensity after 7 days of storage at room temperature. Fixed cells were stored in the below mentioned preservatives. (1) The commercial solution, PreservCyt. Score: + 1 (2) Solution 1, target set on minimum PEG and minimum Sorbitol. Score: + 1 (3) Solution 2, target set on minimum PEG maximum Sorbitol. Score: + 2 (4) Solution 3, target set on maximizing response. Score +2 (5) ladder 10Kb.

The goal, acceptable limit, and importance of each response used for optimization are presented in Table 3. The three resulting optimized solutions were tested in triplicate. The quantitative outcomes for DNA band integrity and DNA concentration are summarized in Table 5. The mean DNA band scores and DNA concentrations were calculated from three independent replicates for each condition. The individual replicates for DNA concentration are shown separately in Table 6. All three formulations exhibited favorable mean DNA band score and concentration compared with PreservCyt; however, the DNA extracted from cells preserved in the solution number 3 displayed superior concentration, and high-intensity band on agarose gel, with semi-quantitative score of 2 in all replicates (Fig 4 and S7 Fig).

**Table 5:** Optimization using the predictive model.

Number	Goal Description	PEG8000	Sorbitol	PH	predicted Score	Predicted Conc.	Desirability%	Lower-Upper Score (CI 95%)	Lower-Upper Conc. (CI 95%)	Actual score	Actual conc.	A260/280	A260/230
**1**	Min PEG & Min Sorbitol	0	1.6	5.6	2.5	214.5	81	2.3-2.7	182-246	1.6	198	1.89	1.85
**2**	Min PEG & Max Sorbitol	0	4.9	5.6	2.0	253.7	96	1.8-2.23	217-290	2	217	1.83	1.96
**3**	Only Max responses	1.2	4.3	5.6	2.2	227.9	100	2.1-2.3	210-246	2	242	1.93	1.89
**PreservCyt**		**–**	**–**	**–**	**–**	**–**	**–**	**–**	**–**	1.3	180	1.92	1.93

**Table 6:** DNA concentration replicates and summary statistics for optimized fixatives and PreservCyt.

Condition	Rep 1	Rep 2	Rep 3	Mean	SD	CV%
**Solution 1**	180	200	214	198	17.1	8.6%
**Solution 2**	208	217	226	217	9.0	4.1%
**Solution 3**	230	244	252	242	11.1	4.6%
**PreservCyt**	170	182	188	180	9.17	5.1%

**Fig 5:**
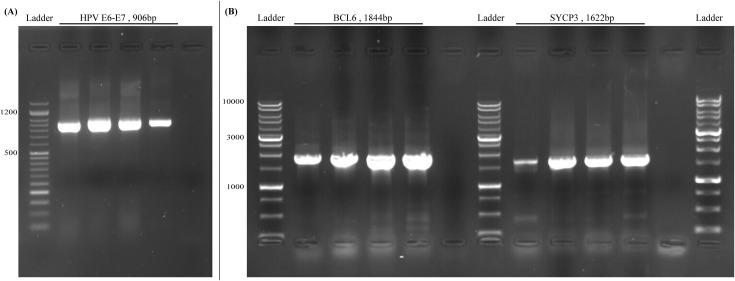
PCR products of final solutions. **(A)** The forward primer was designed to target the integrated HPV 18 genome within the *E6* gene. The reverse primer, on the other hand, was positioned within the *E7* gene, resulting in the amplification of a DNA fragment approximately 906 base pairs in length. A replicate experiment is provided in S8 Fig. **(B)** PCR amplification of two assessed fragments related to the *BCL6* and *SYCP3* yielded robust products without inhibition, confirming the suitability of the DNA for molecular diagnostics. Amplified products were respectively approximately 1844 and 1622 bp in length, as expected.

To further investigate the integrity of stored DNA within 7 days, three sets of fragments were amplified using the primer pairs targeting a 1844 bp-fragment of *BCL6* gene, a 1622 bp-fragment of *SYCP3* gene, and a 906 bp-fragment on *E6/E7* junction on DNA. In all cases the desired fragment was successfully amplified (Fig 5).

Among the three optimal formulations, one that stood out contained 40% ethanol, 4.27% Sorbitol, 1.17% PEG 8000, and a pH of 5.6. This combination not only performed better than the commercial PreservCyt solution but also was cost-effective.

The Table 5 represents the predicted and actual experimental responses for three optimized solutions. The desirability score of all 3 conditions is satisfactory. The consistency between predicted values and experimental results indicates that the ANOVA-based model created by Design-Expert effectively reflects these outcomes. The tight 95% confidence intervals further suggest low variability and precise model predictions.

### 3.4 RNA integrity assessment

**Fig 6:**
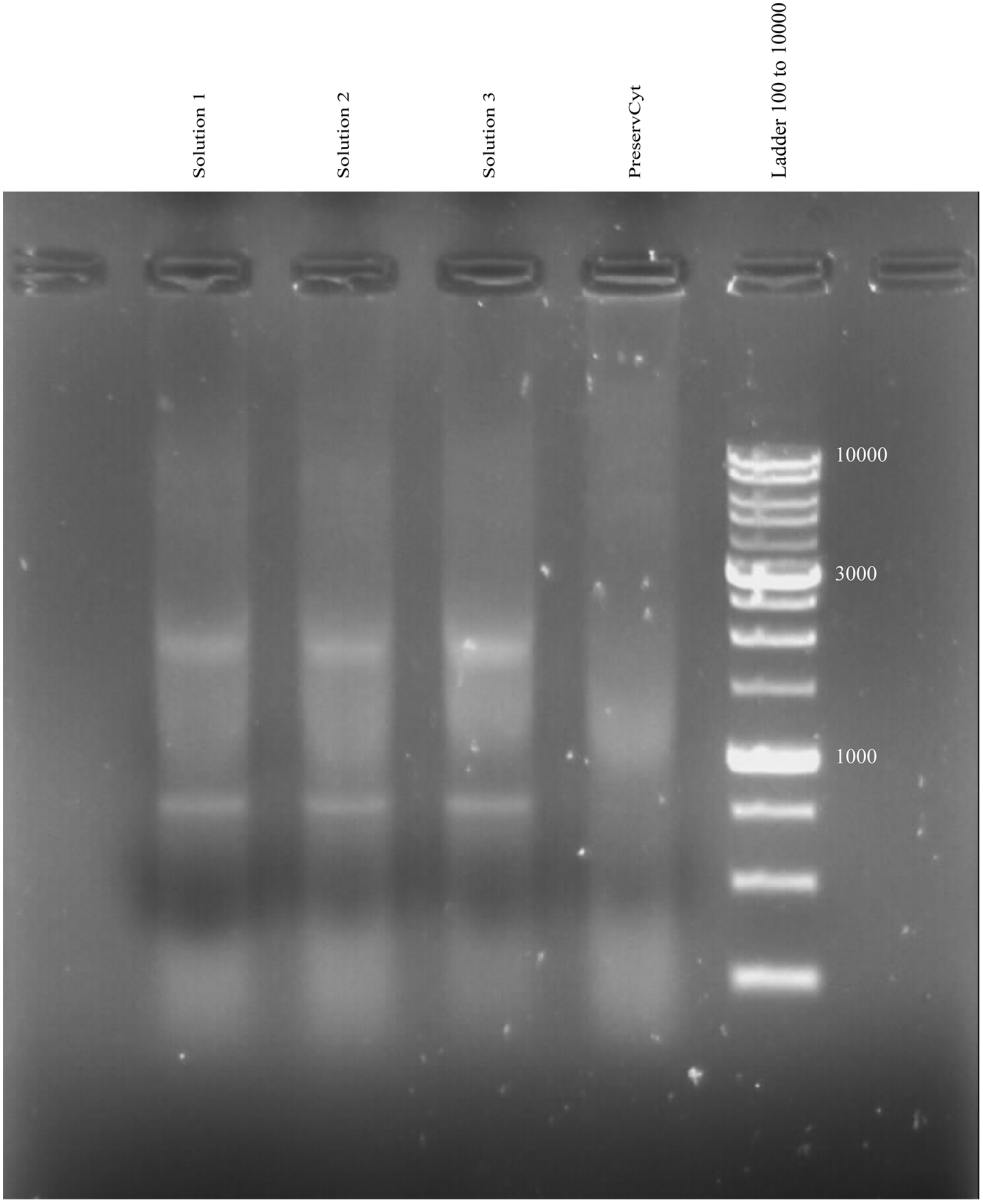
Comparing RNA integrity for final solutions and standard. Total RNA extracted from stored cells in three suggested solutions for optimizing nucleic acid preservation is superior to a commercial solution on 1% Agarose Gel. (1) solution 1. (2) solution 2. (3) solution 3. (4) the commercial solution, PreservCyt.

**Fig 7:**
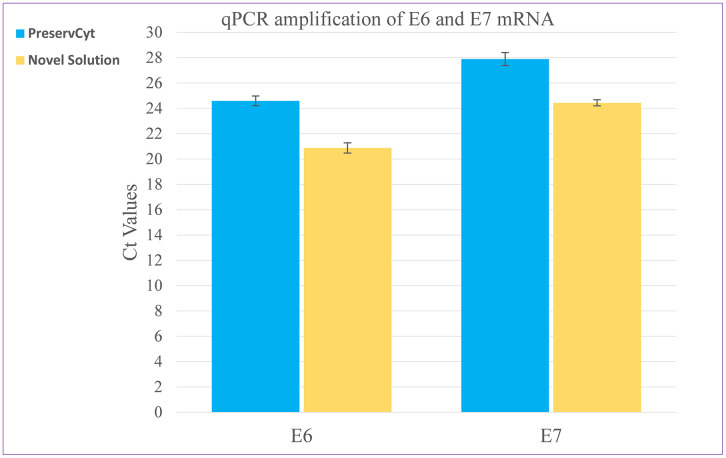
qRT-PCR on E6 and E7 mRNA. Comparing Ct values between the optimized fixative and commercial one when stored at room temperature, the integrated HPV18 *E6* and *E7* genes within the HeLa transcriptome remained more intact in the novel fixative for up to 7 days. Each data point presented represents the mean Ct from triplicate reactions.

RNA quality was maintained in the three suggested fixatives after optimization step. Integrity was indicated by distinct 28S and 18S rRNA bands on agarose gel (Fig 6). Expected pattern of distinct rRNA bands is observed in positive control, while no bands were detected in the negative control (S9 Fig). Real-time PCR amplification was performed on the final novel solution that was suggested by software based on maximizing responses. This novel formula contained 4.27% Sorbitol and 1.17% PEG 8000. Amplification of the *E6* and *E7* oncogenes demonstrated lower Ct values for the final optimized fixative compared to PreservCyt, indicating superior RNA quality and preservation (Fig 7, S3 Table). Amplification curve of E6 and E7 genes are provided in S10 Fig and S11 Fig.

### 3.5 Comparison of morphological preservation

**Fig 8:**
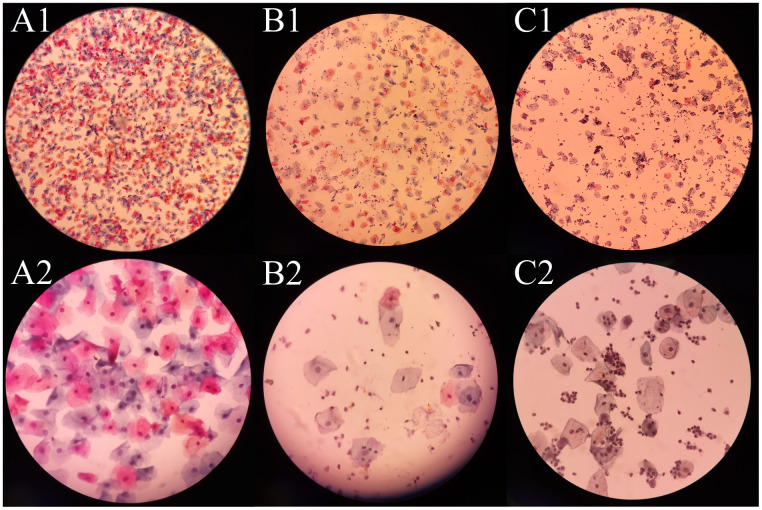
Clinical samples. Three matched slide pairs from patient cervical swabs were evaluated using blinded, quantitative scoring. The top and bottom panels in Fig 8 correspond to the same panels in Fig 9, shown in the same order. Top panels are shown at 100 × magnification, and bottom panels at 400 × magnification.

**Fig 9:**
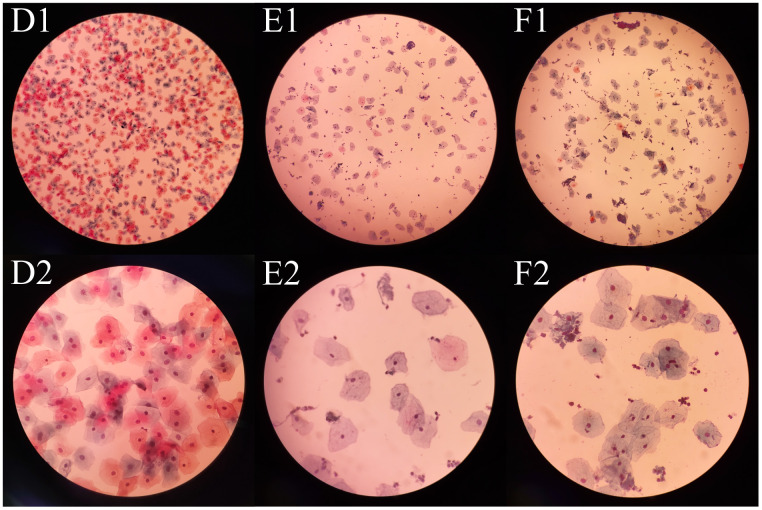
Clinical samples. Three matched slide pairs from patient cervical swabs were evaluated using blinded, quantitative scoring. The top and bottom panels in Fig 8 correspond to the same panels in Fig 9, shown in the same order. Top panels are shown at 100 × magnification, and bottom panels at 400 × magnification.

Blinded ordinal scoring demonstrated high concordance between cytopathologists. Across the three matched pairs used for inter-rater evaluation, agreement ranged from 95.3% to 98.0%. Standard deviations of individual rater scores were low (0.083–0.255), and all values fell within their corresponding Mean ± 3SD limits, indicating stable and internally consistent scoring behavior. Paired comparisons of averaged feature scores showed comparable cytomorphological quality between the Novel fixative and PreservCyt® (Figs 8 and 9, and Table 7). Statistical analysis for three paired slides is provided in supplementary S4 Table.

**Table 7:** Blinded ordinal scoring by two pathologists for three paired slides.

Pair	Rater	Cell Type Representation	Staining Clarity	Cell Distribution	Background Cleanliness	Morphologic Preservation	Pair Mean	SD	Mean – 3SD	Mean + 3SD	Pair Mean Agreement (%)
1 (A/D)	R1	2.8	2.8	2.9	2.6	2.9	2.80	0.12	2.43	3.17	95.3
	R2	3.0	3.0	2.8	2.7	3.0	2.90	0.1	2.48	3.32	
2 (B/E)	R1	3.0	2.8	3.0	2.7	2.8	2.86	0.13	2.46	3.26	96.0
	R2	2.8	2.7	2.9	2.8	2.7	2.78	0.08	2.53	3.03	
3 (C/F)	R1	2.6	2.5	2.4	2.2	2.6	2.46	0.17	1.96	2.96	98.0
	R2	2.6	2.5	2.3	2.0	2.6	2.40	0.25	1.63	3.16	

Scores from the five fields are averaged per feature. One mean score per feature per slide per rater (pathologist) is provided.

### 3.6 HPV genotyping by DNA analysis

As shown in Table 8, HPV DNA was detected in all seven samples from the symptomatic or previously HPV-positive group, while all seven samples from participants with no known risk factors tested negative. Among the positive samples, high-risk HPV genotypes were identified in four samples (16, 18, 35, and 51), and low-risk genotypes in four samples (42, 43, 54, and 62). Some samples contained both high- and low-risk HPV types. Representative real-time PCR amplification curves confirming HPV genotyping are provided in S2 File. These results demonstrate that the novel fixative preserves DNA quality sufficient for accurate HPV genotyping.

**Table 8:** HPV Real Time PCR on vaginal discharge.

Sample ID	Risk Group	HPV Result	Detected Genotypes (High Risk/Low Risk)
11829	Symptomatic/Previous positive	Positive	35HR/54LR
11865	Symptomatic/Previous positive	Positive	18HR/-
11866	Symptomatic/Previous positive	Positive	16HR/-
11869	Symptomatic/Previous positive	Positive	-/62LR
11874	Symptomatic/Previous positive	Positive	-/62LR
11876	Symptomatic/Previous positive	Positive	-/42LR, 43LR
11877	Symptomatic/Previous positive	Positive	51HR/-
11863	No risk	Negative	–
11870	No risk	Negative	–
11898	No risk	Negative	–
11859	No risk	Negative	–
11897	No risk	Negative	–
11910	No risk	Negative	–
11912	No risk	Negative	–

## 4. Discussion

Cervical cancer can be preventable. Liquid-based cytology is a common HPV screening method defined as transferring the cervical specimens to a liquid media that preserves the samples for following diagnostic tests [18]. The majority of cervical cancer cases are caused by human papillomavirus (HPV) and most of them occur in developing countries [19]. Recent updates in HPV screening guidelines include E6/E7 mRNA test that qualifies viral mRNA load for14 high-risk types [20].

Understanding the significance of preservatives and how they affect the molecular profile of biological specimens is crucial to addressing the degradation processes that start as soon as a sample is taken from a patient and continue during processing if improperly managed [21,22]. The preservation of the quality of liquid-based preparation (LBP) sample even after cytopathologic diagnosis, can be a useful source of nucleic acids for further molecular diagnostics, however RNA preservation in these samples is challenging [23]. This study considered environmentally friendly material for developing a new cell preservative designed to maintain cell morphology, DNA integrity, and RNA integrity at room temperature.

In our search for biodegradable options, we have taken into account ingredients that are frequently found in alcohol-based preservatives, such as ethanol, polyethylene glycols as well as sugars [24,25]. We investigated how polyols, such as Sorbitol and polyethylene glycol (PEG), interact with nucleic acids over extended periods at room temperature using a well-structured design. PEG has various molecular weights, but those with similar physicochemical properties tend to share similar applications. In our research, we used PEG 8000 based on its availability and unpublished earlier results that showed PEG 8000 plays a role in our study. Some relevant characteristics have been reported for PEG 8000. It has been successfully used as a cryoprotective agent that helps maintaining membrane integrity during cryopreservation and acts like an osmotic protector [26]. Besides PEG 8000 has a crowding effect that prevents cell clumping or improves the separation of particles that is useful for single-cell RNA analysis [27]. These properties can also be beneficial for liquid-based cytology.

According to our study, Sorbitol content, pH, and PEG concentration all have considerable effects on the stability of nucleic acids over time. With 40% ethanol, 4.26% Sorbitol, 1.17% PEG 8000, and a pH of 5.6, we created a novel ethanol-based fixative that provided nucleic acid preservation. Our results suggest that the concentration of PEG should be kept to a minimum, although higher concentrations of sorbitol are more beneficial for preservation. We also observed that approximately 5% PEG 8000 (S1 Table) reduced DNA yield during extraction. This finding aligns with the previous research on membrane stability, which showed that high concentrations of higher molecular weight PEG decreased membrane integrity [28]. According to earlier studies, RNA from new coronavirus samples can be successfully preserved for up to 10 days at room temperature using Sorbitol concentrations ranging from 0.5% to 5% [29].

Citric acid was included in the final formulation as a chelating agent and improved the stability of DNA and RNA by reducing nuclease activity. Besides being a chelate for di-cationic ions, citric acid whose structure is comparable to that of small acidic peptides, like Dnase2 [30] can bind to DNA in the presence of divalent cations (Cu2 + , Fe2 + , Zn2 + , Mg2+) [31]; By these actions citric acid probably limits activity and accession of DNase to DNA. The concentration of ions such as Na + , K + , Mg2 + , and Ca2+ in the buffer affects RNase activity [32]. As a result, RNase activity can be controlled by storage media composition, which impacts RNA integrity. rRNA is a good indicator of overall RNA integrity since it is more prone to degradation than other forms of RNA [32]. We discovered that a solution comprising 10 mM acetate buffer, 5mM citric acid, 40% ethanol, 4.3% sorbitol, and 1.2% PEG was more successful in maintaining RNA integrity compared to the standard PreservCyt solution.

Polyols like Sorbitol function as compatible solutes, helping to maintain cellular integrity under stress conditions [33]. PEG and Mannitol as polyols are used in biological specimen preservative and may protect nucleic acids by stabilizing their structure and preventing enzymatic degradation (WO2011103114A1) [34]. These substances could decrease the negative impact of environmental conditions on the stability of nucleic acids while they are being stored. PEG 8000 and Sorbitol likely act as crowding agents [35], modifying hydrogen bonds within and between biomolecules, which leads to modulation of enzyme activity [36]. In addition, the chemical relationship between Sorbitol and PEG revealed how important it is to balance their concentrations for optimal preservation.

While the addition of PEG8000 and sorbitol effectively preserved cellular morphology and supported nucleic acid integrity over seven days of storage at room temperature, the potential impact of this formulation on native protein conformation and epitope stability remains to be determined. Also compatibility with assays such as immunohistochemistry (IHC) remains unclear and requires further investigation.

The performance of the novel fixative was evaluated using HeLa cell line and cervical samples. Clinical specimens were analyzed by both cytological and molecular methods. Morphological analyses in this study were conducted taking two approaches. First, a general similarity assessment was performed on three cervical specimens preserved in the novel fixative and in the standard PreservCyt solution. Slides were evaluated by a pathologist blinded to the preservation method. This preliminary analysis was intended to confirm the feasibility of morphological preservation. Preliminary morphology characterization data are provided in S3 File. The second approach expanded the clinical arm and employed a blinded ordinal scoring rubric with assessment of inter-rater agreement. Fourteen clinical samples were collected for molecular analysis, and three of them were divided in two and processed in parallel using both fixatives for detailed cytological comparison by two blinded cytopathologists. Statistical analysis demonstrated agreement and reproducibility. This progression from initial morphological observation to quantitative analysis supports the conclusion that the novel fixative preserves cytomorphology comparably to the standard reference.

Our results suggest that the optimized formulation maintains clear genomic DNA bands and supports RNA integrity after seven days of storage at room temperature. Semi-quantitative scoring and effective amplification of HPV-18 oncogenes (*E6* and *E7*), without PCR inhibition, further support the DNA’s integrity. In addition, HPV DNA was successfully amplified from 14 cervical samples, detecting both high-risk (16, 18, 35, 51) and low-risk (42, 43, 54, 62) genotypes, while samples from participants with no known risk factors tested negative. These results demonstrate that the novel fixative supports accurate HPV genotyping and reliable molecular diagnostics.

RNA quality was also preserved by the existence of 28S and 18S rRNA bands on an agarose gel and low Ct values in qRT-PCR, suggesting minimal degradation. These findings suggest that the proposed fixative may be suitable for nucleic acid–based diagnostics, particularly in resource-constrained settings where cold storage is not feasible. Although polyols are shown to act as stabilizing agents, the extent to which they preserve nucleic acid integrity across different conditions and longer storage periods remains to be fully characterized. Nucleic acid preservation should be evaluated by advanced molecular integrity metrics such as DNA Integrity Number (DIN) and RNA Integrity Number (RIN) in future studies.

The fixatives that are currently on the market, like methanol-based solutions [12], frequently present safety and environmental risks in addition to not effectively preserving RNA [37]. Ethanol-based solutions are typically safer, but in order to achieve better results, their formulations need to be precisely optimized. We filled a gap by systematically evaluating the impact of pH changes and environmentally friendly polyhydric chemicals on nucleic acid preservation. The ability to store nucleic acids at ambient temperature while maintaining quality has important implications for molecular diagnostics. Our new fixative targets HPV testing, aiming to improve diagnosis and prognosis of cervical cancer; however future studies using larger sample sizes and more advanced molecular analyses including DIN, RIN, and proteomic assessments, are required to further evaluate the performance of the proposed fixative.

In summary, our study presents a new formulation for HPV and LBC screening that may offer a cost-effective and environmentally safer alternative. Our fixative appeared to preserve both nucleic acid integrity and cellular morphology, as revealed by Pap-stained slides of cervical exfoliated cells and HPV genotyping by Real Time PCR, which may support both molecular and morphological diagnostic requirements.

## Supporting information

S1 Table Response values for designed experiments per two blocks. 48 experimental Runs plus two basal conditions.
(XLSX)

S2 Table Twelve zero Runs analysis. One factor (either PEG or Sorbitol) was completely omitted (0%), while the other was maintained at its center level. They were compared with the two basal combinations that did not include any additives having two PH levels. Based on the mean intensity scores of DNA bands and the mean DNA concentrations measured by absorbance at 260 nm, the addition of both polyols, PEG and sorbitol, improved preservation. However, sorbitol appeared to have a greater influence on the DNA stability.
(XLSX)

S3 Table qPCR amplification of E6 and E7 mRNA. Data for all three replicates is provided.
(XLSX)

S4 Table Pairwise cytomorphology scores. (XLSX)

S1 File Raw images of main body gels. (PDF)

S2 File HPV Genotyping amplification curve. (PDF)

S3 File Preliminary morphology characterization. (PDF)

S1 Fig Raw gel image corresponding to Fig 1. The upper lanes are cropped out and reported in manuscript Fig 1. The lower lanes represent (left to right): 1- basal solution with no polyol compounds 2-addition of 2% PEG 3-addition of 2% Sorbitol 4- addition of 1% Sorbitol and 1% PEG.
(TIF)

S2 Fig Raw gel image corresponding to Fig 2. (TIF)

S3 Fig Raw gel image corresponding to Fig 4. The five right lanes from the top row were cropped and presented in Fig 4. The remaining lanes represent genomic DNA from the experimental runs within the design space.
(TIF)

S4 Fig Raw gel image corresponding to Fig 5. The five right-most lanes were cropped and presented in Fig 5, while the remaining lanes are not related to this study.
(TIF)

S5 Fig Raw gel image corresponding to Fig 5. The top row was cropped and presented in Fig 5, while the bottom row shows the E6–E7 oncogenes of HPV, yielding a product size of 906 bp for the experimental runs within the design space.
(TIF)

S6 Fig Raw gel image corresponding to Fig 6. (TIF)

S7 Fig Replication of Final solutions vs. standard. Genomic DNA bands with different intensity after 7 days of storage at room temperature. 1- basal solution with no polyol compounds, Score: + 1, 2- Solution 1, Score: + 2, 3- Replication of basal solution, Score: 0, 4- Solution 2, Score: + 2, 5- PreservCyt, Score: + 1, 6- Solution 3, Score: + 2.
(TIF)

S8 Fig Replication of HPV amplification on Final solutions vs. standard. 906 bp PCR products of HPV E6–E7 oncogenes for the three optimized solutions and PreservCyt.
(TIF)

S9 Fig Positive and Negative Control of RNA integrity. RNA Integrity on Agarose Gel is indicated: 1- HeLa cells suspended in PBS (7 days’ storage at RT) 2- HeLa cells preserved in the reference fixative, PreservCyt (7 days’ storage at RT) 3- Positive Control- RNA of Fresh HeLa cells extracted using TRIzol reagent 4- Negative control: loading dye and water without RNA.
(TIF)

S10 Fig Amplification curve of E6. (TIFF)

S11 Fig Amplification curve of E7. (TIFF)
