# Supplementary figures and images for: Optimizing an ethanol-based fixative for enhanced nucleic acid preservation in cervical samples using a central composite design approach
Source: PLoS One. 2026 Jun 26;21(6):e0349088. doi: 10.1371/journal.pone.0349088 (PMC13308814; doi:10.1371/journal.pone.0349088)

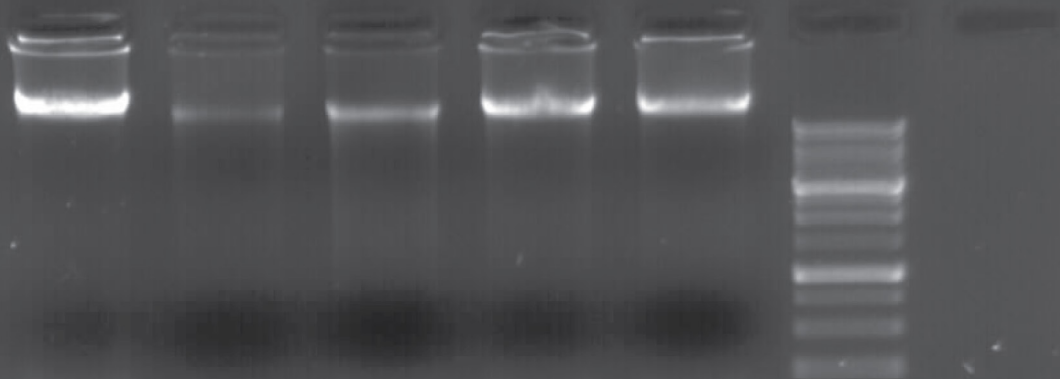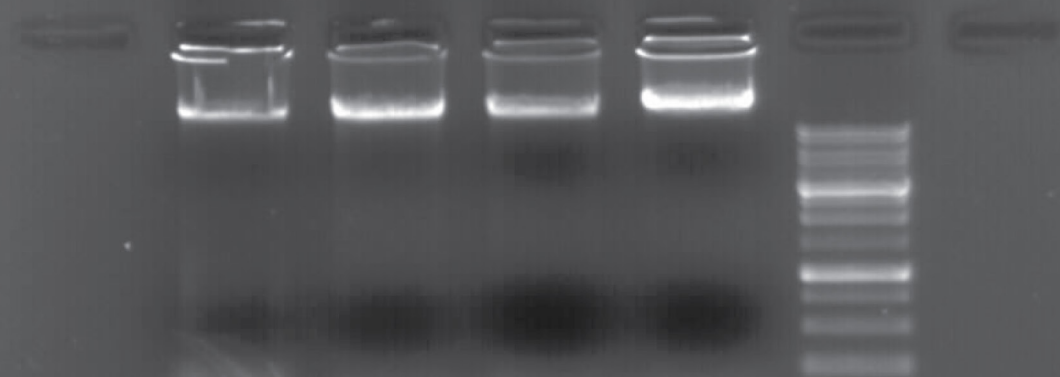

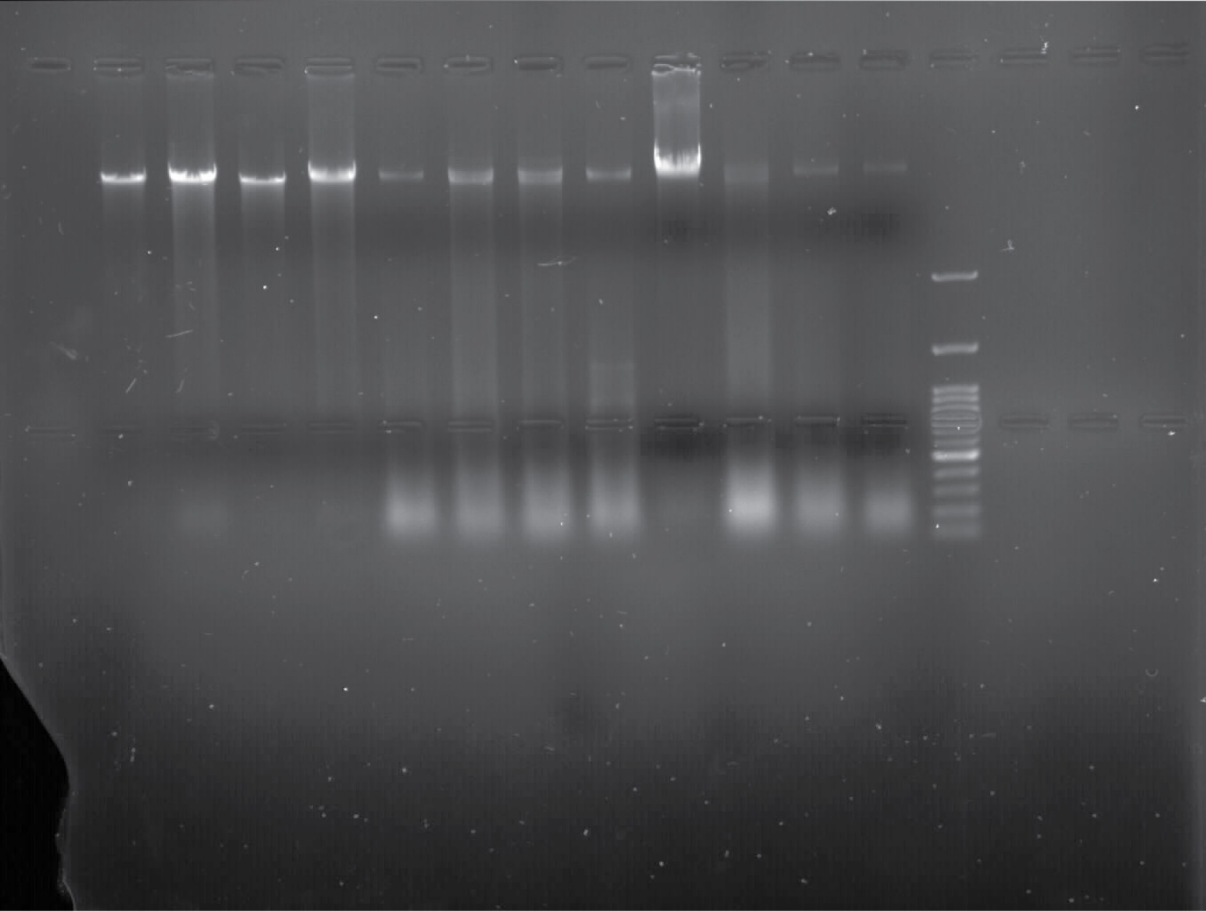

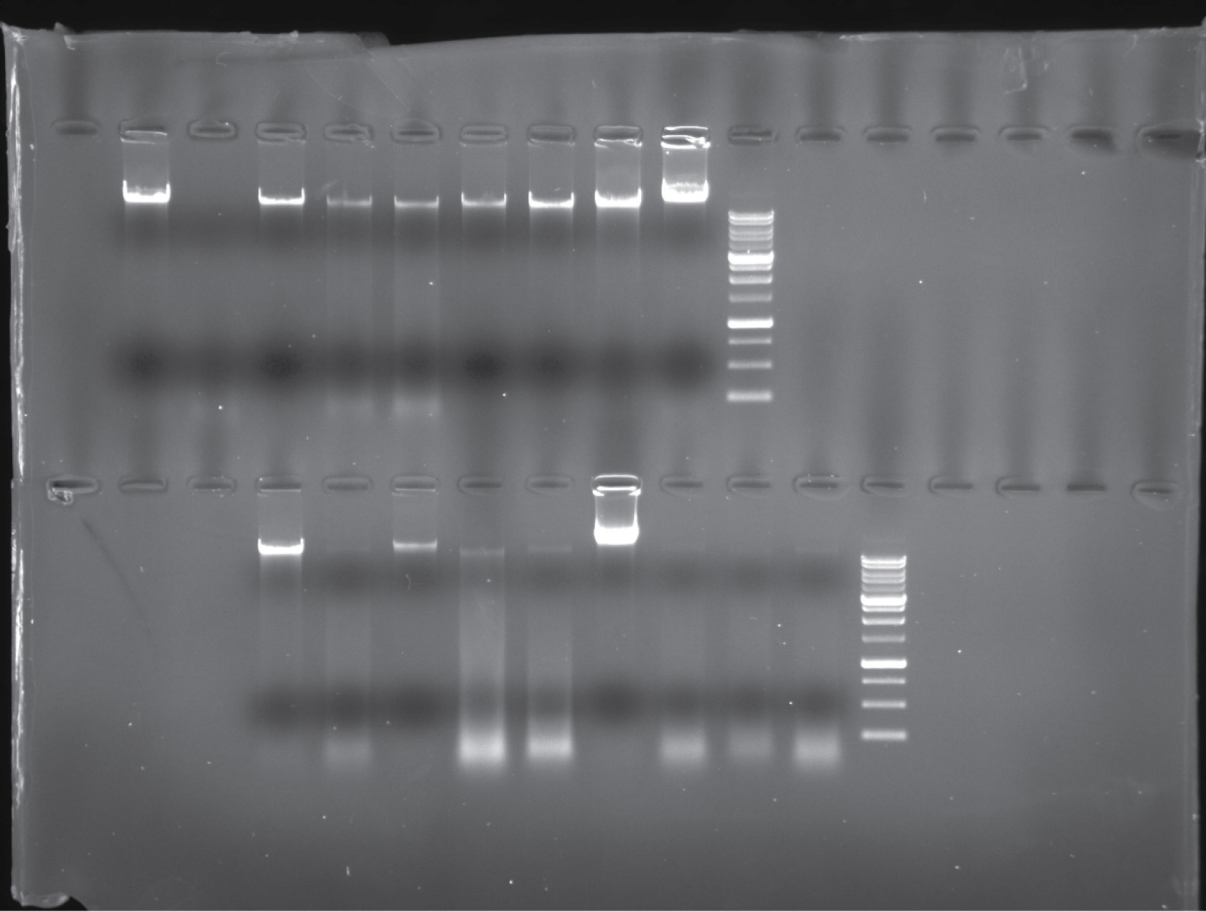

1 2 3 4 5 6 7 8 9 10 11 12 13 14 15 16 17 18 19 20

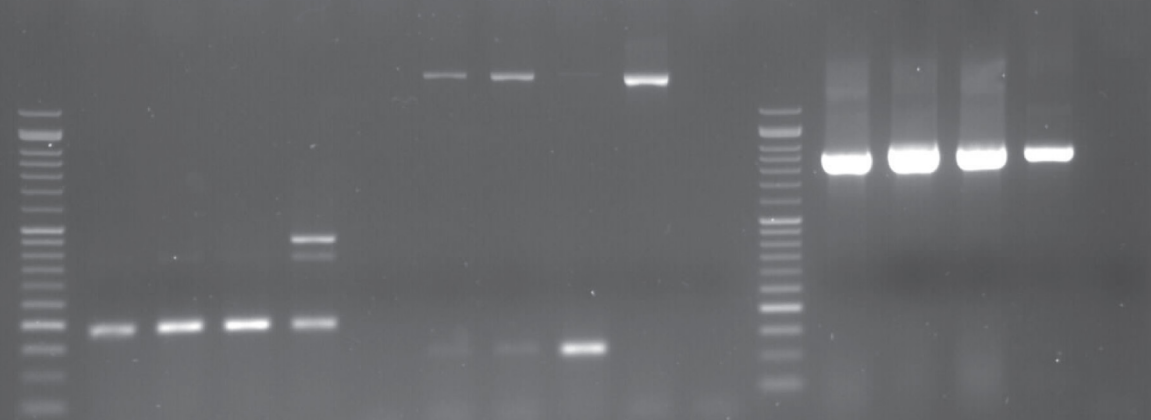

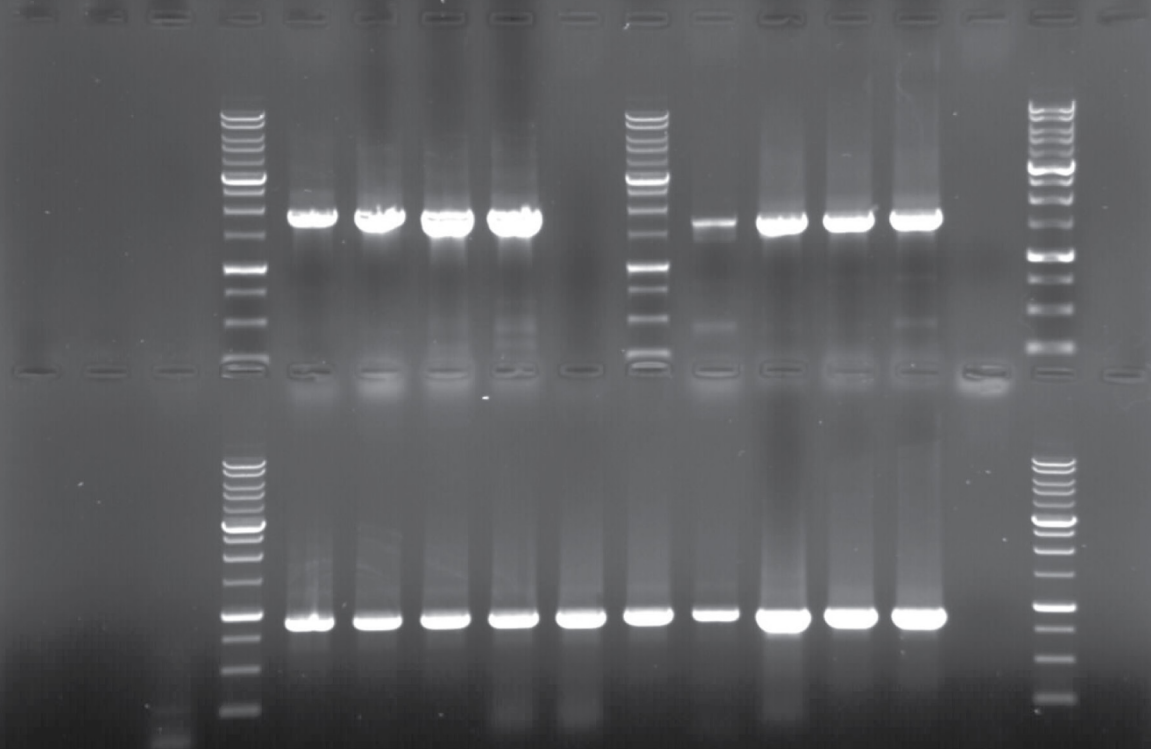

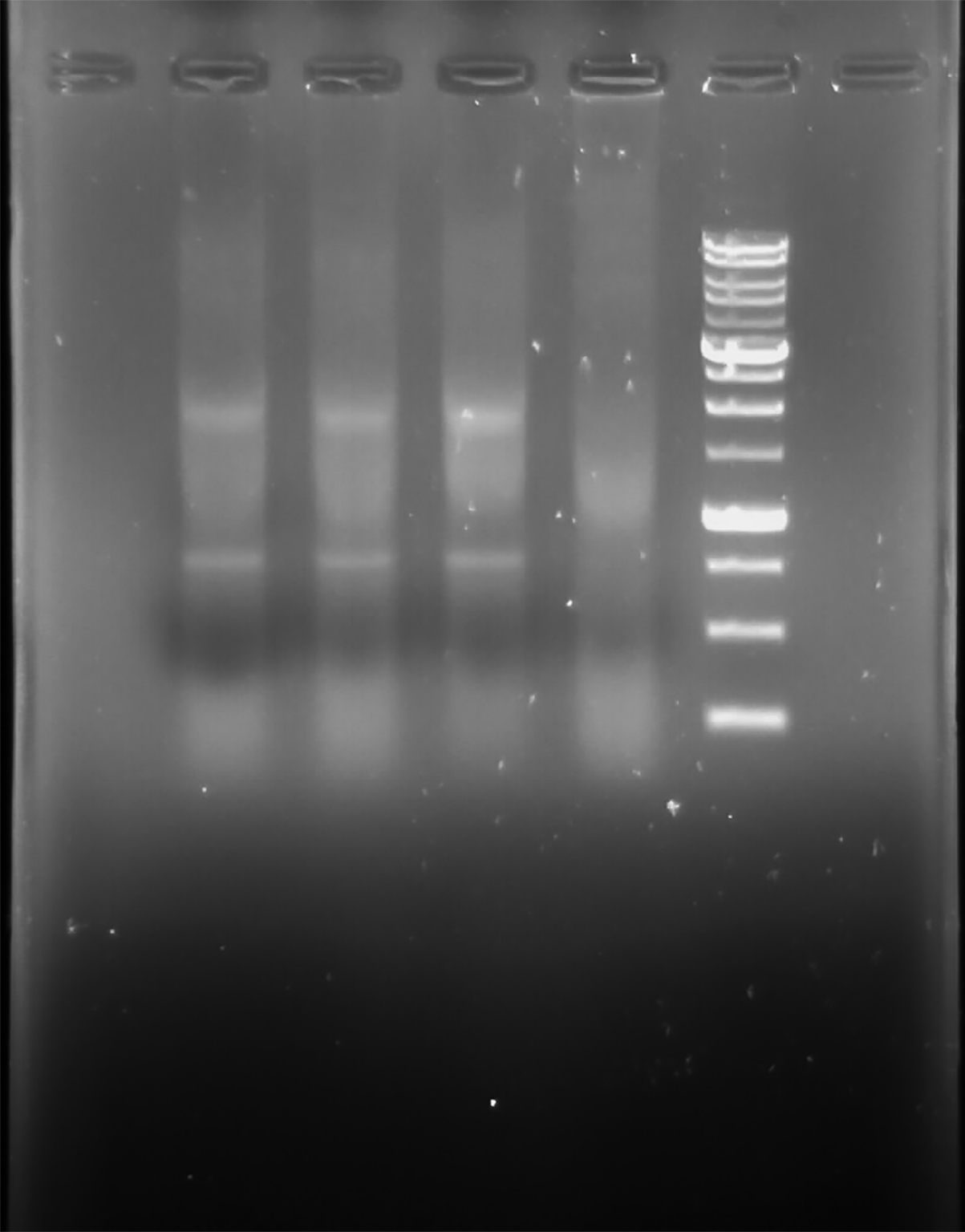

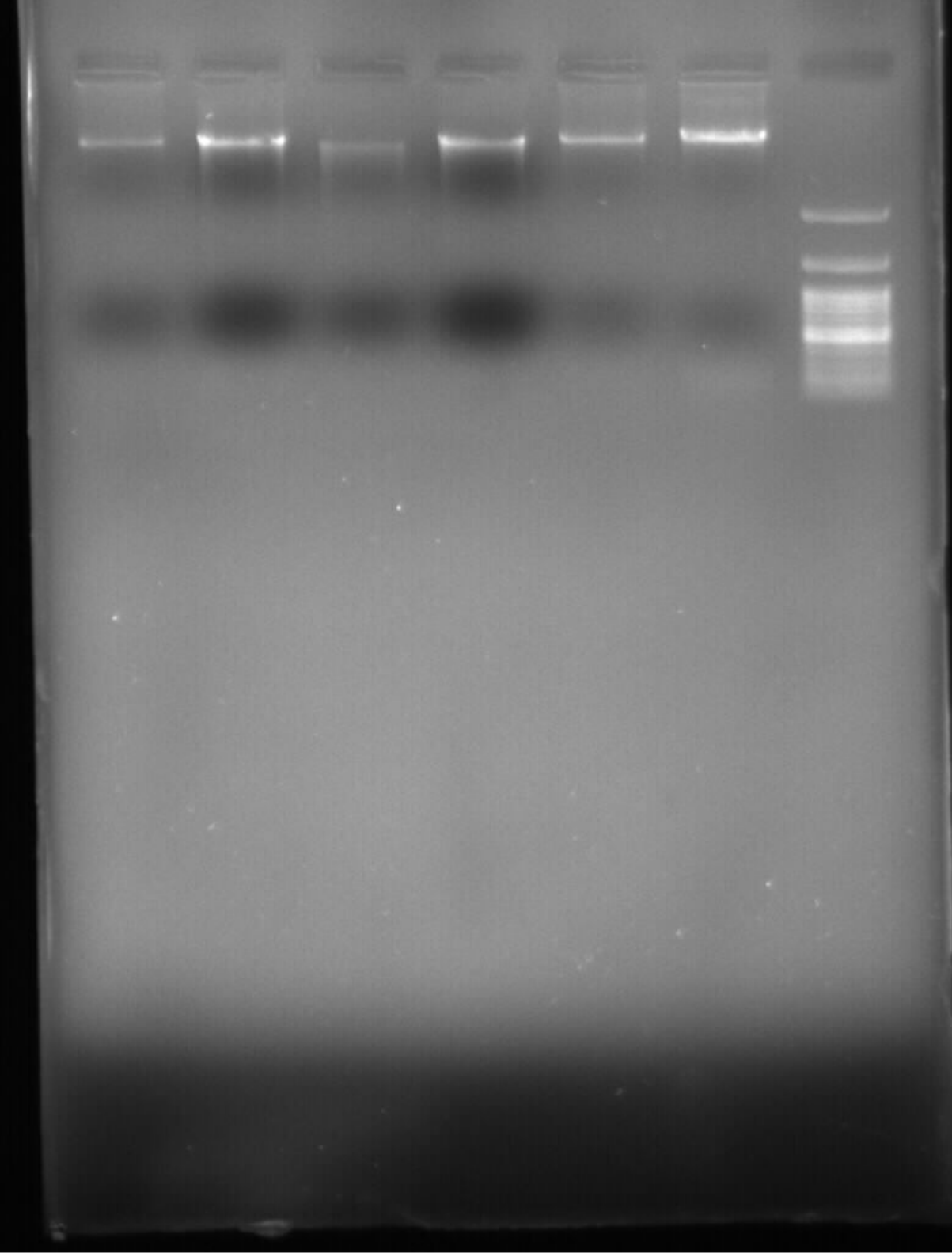

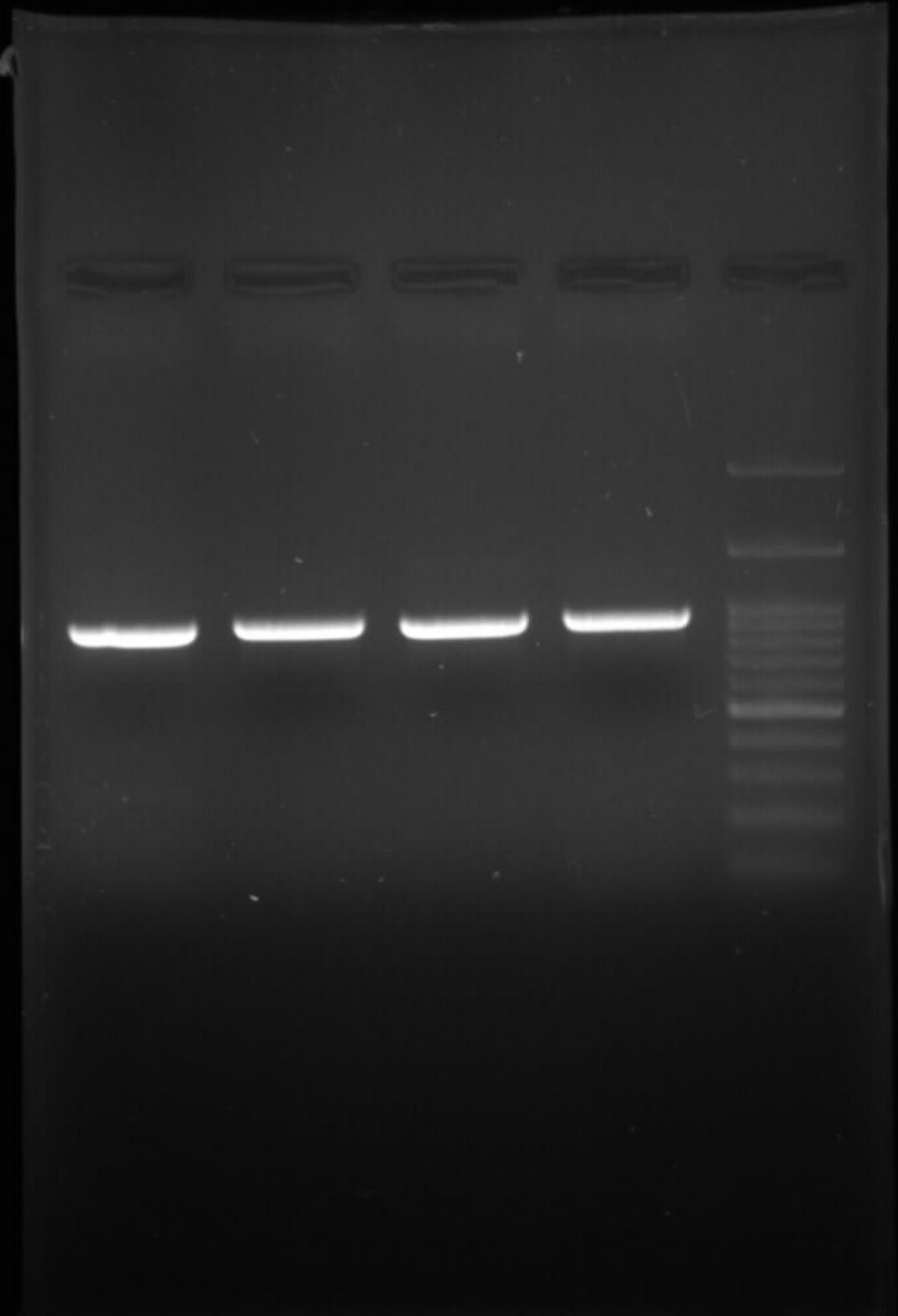

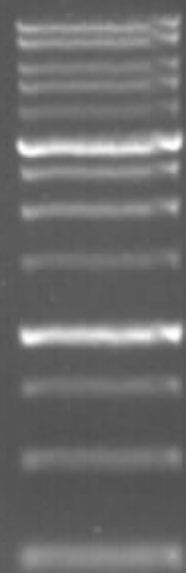

Supplement: S1 File — (PDF) [file pone.0349088.s005.pdf]

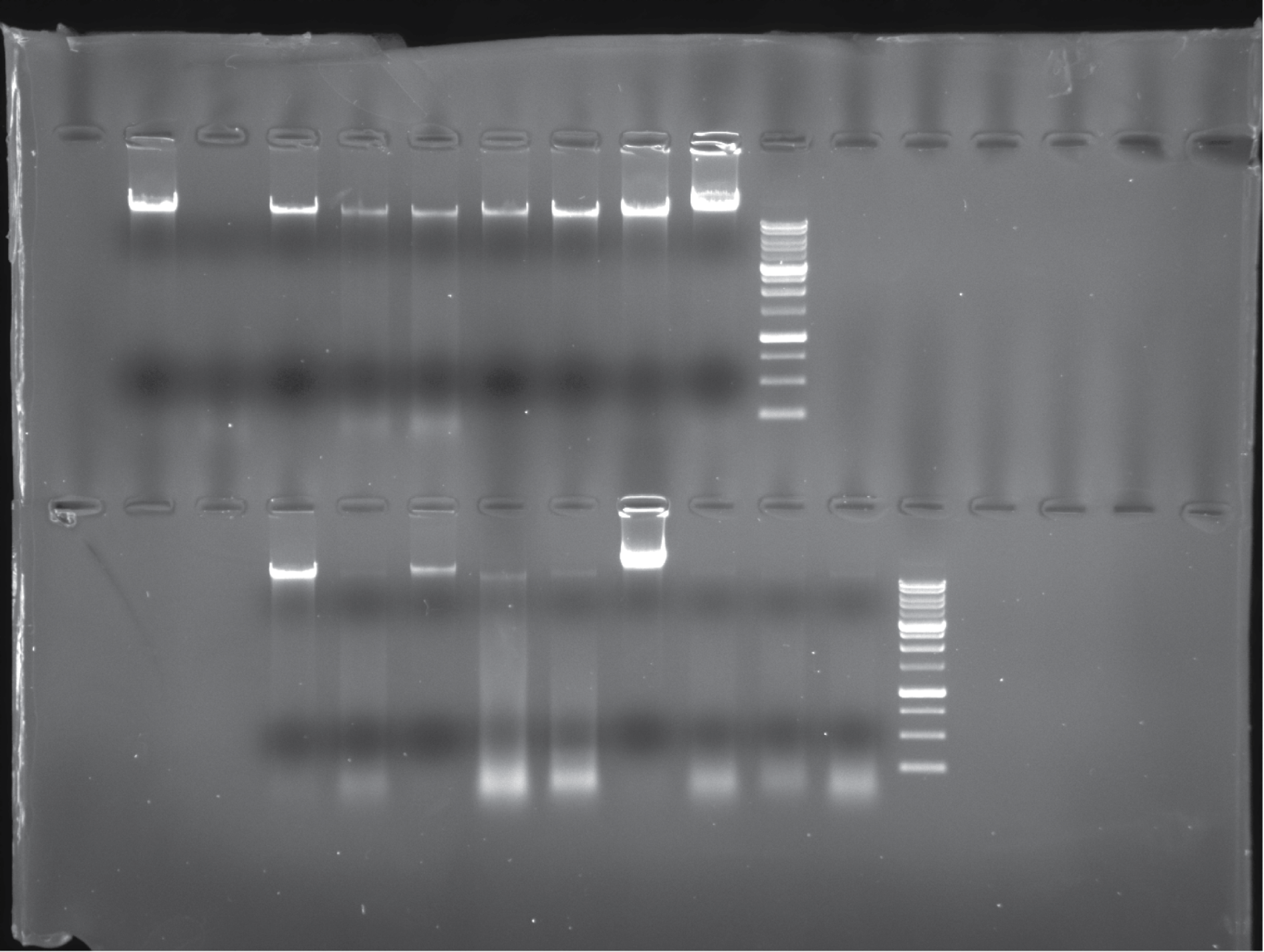

Supplement: S1 Fig — The upper lanes are cropped out and reported in manuscript Fig 1. The lower lanes represent (left to right): 1- basal solution with no polyol compounds 2-addition of 2% PEG 3-addition of 2% Sorbitol 4- addition of 1% Sorbitol and 1% PEG. (TIF) [file pone.0349088.s010.tif]

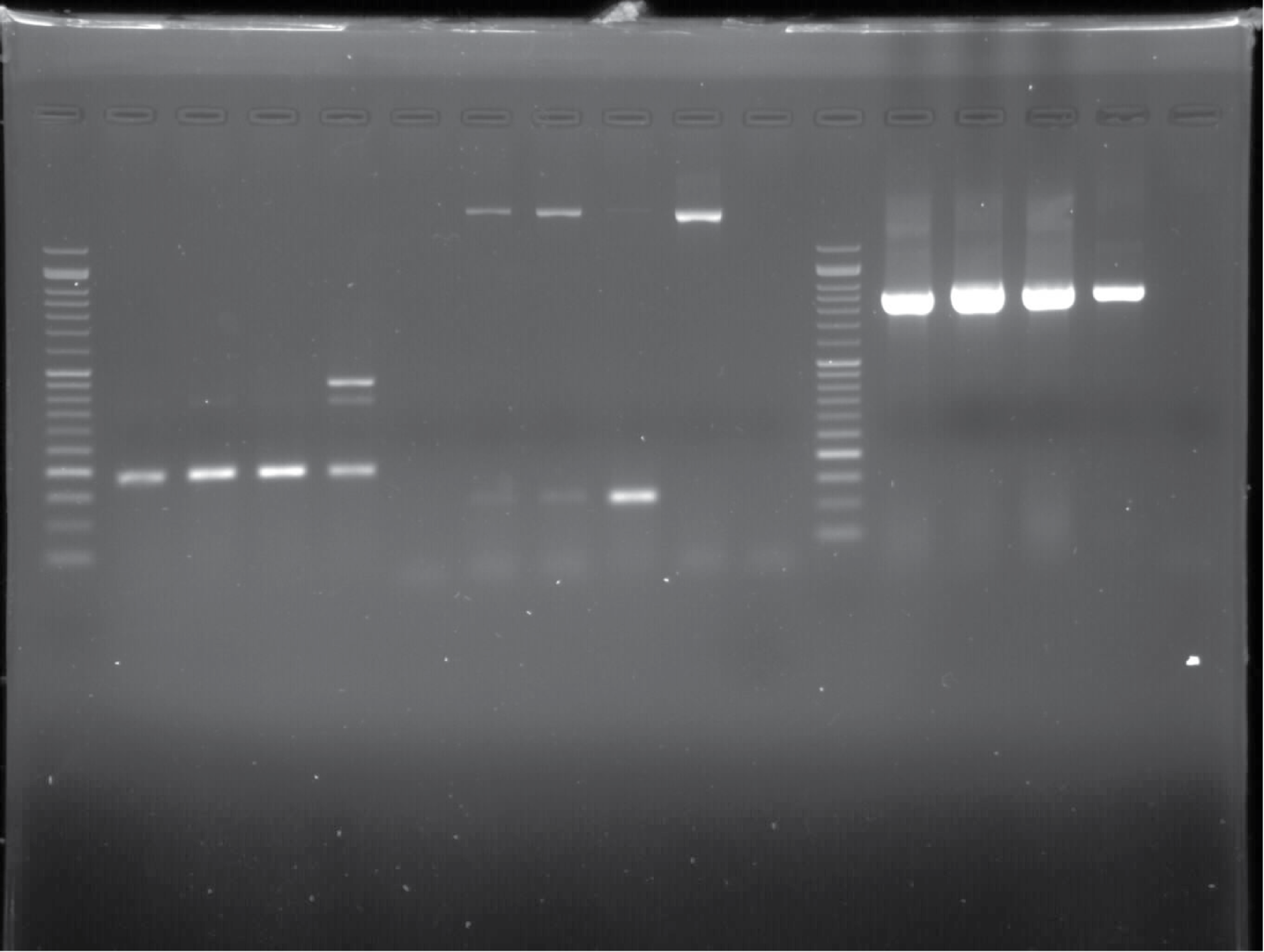

Supplement: S2 Fig — (TIF) [file pone.0349088.s011.tif]

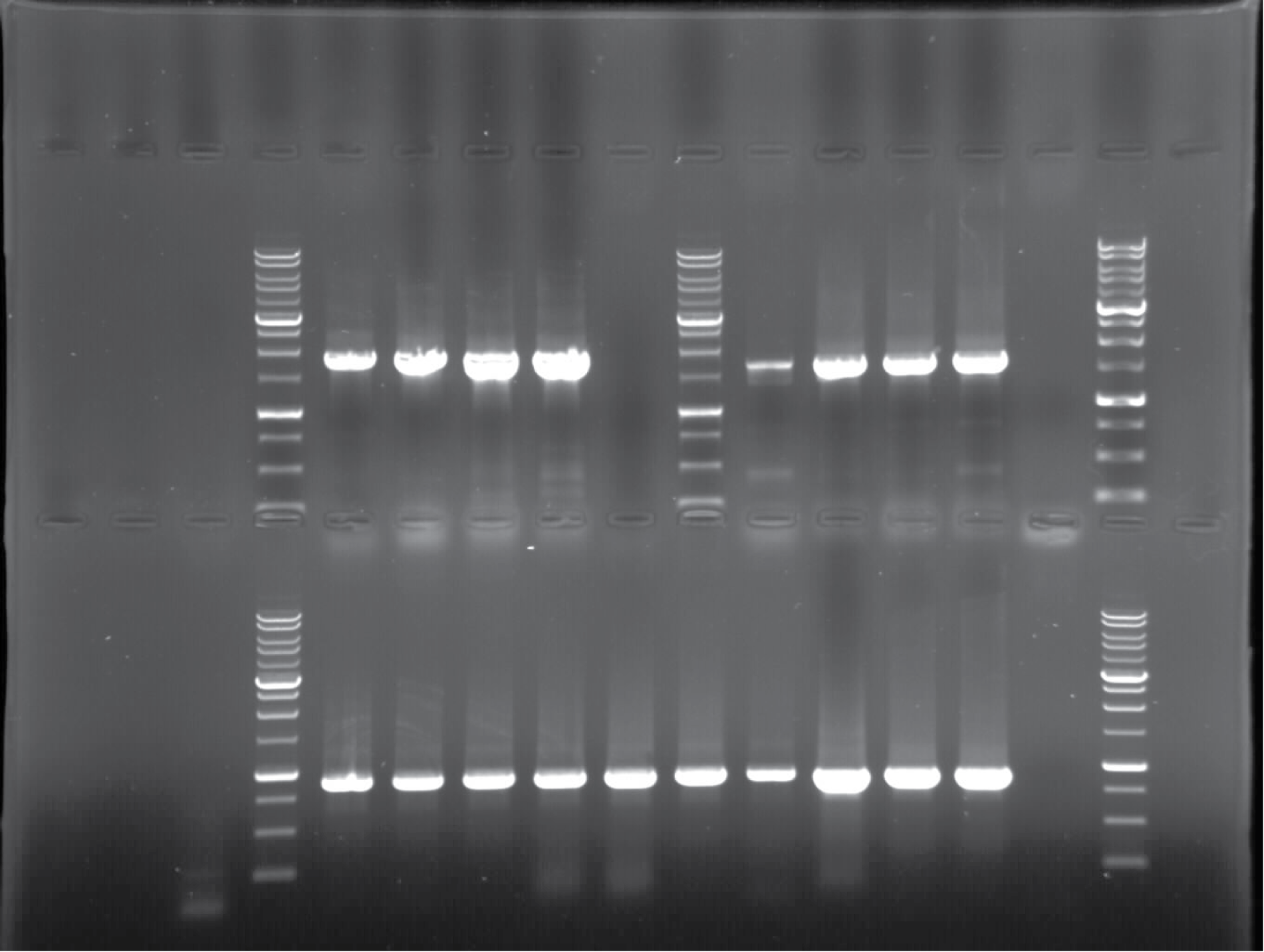

Supplement: S3 Fig — The five right lanes from the top row were cropped and presented in Fig 4. The remaining lanes represent genomic DNA from the experimental runs within the design space. (TIF) [file pone.0349088.s012.tif]

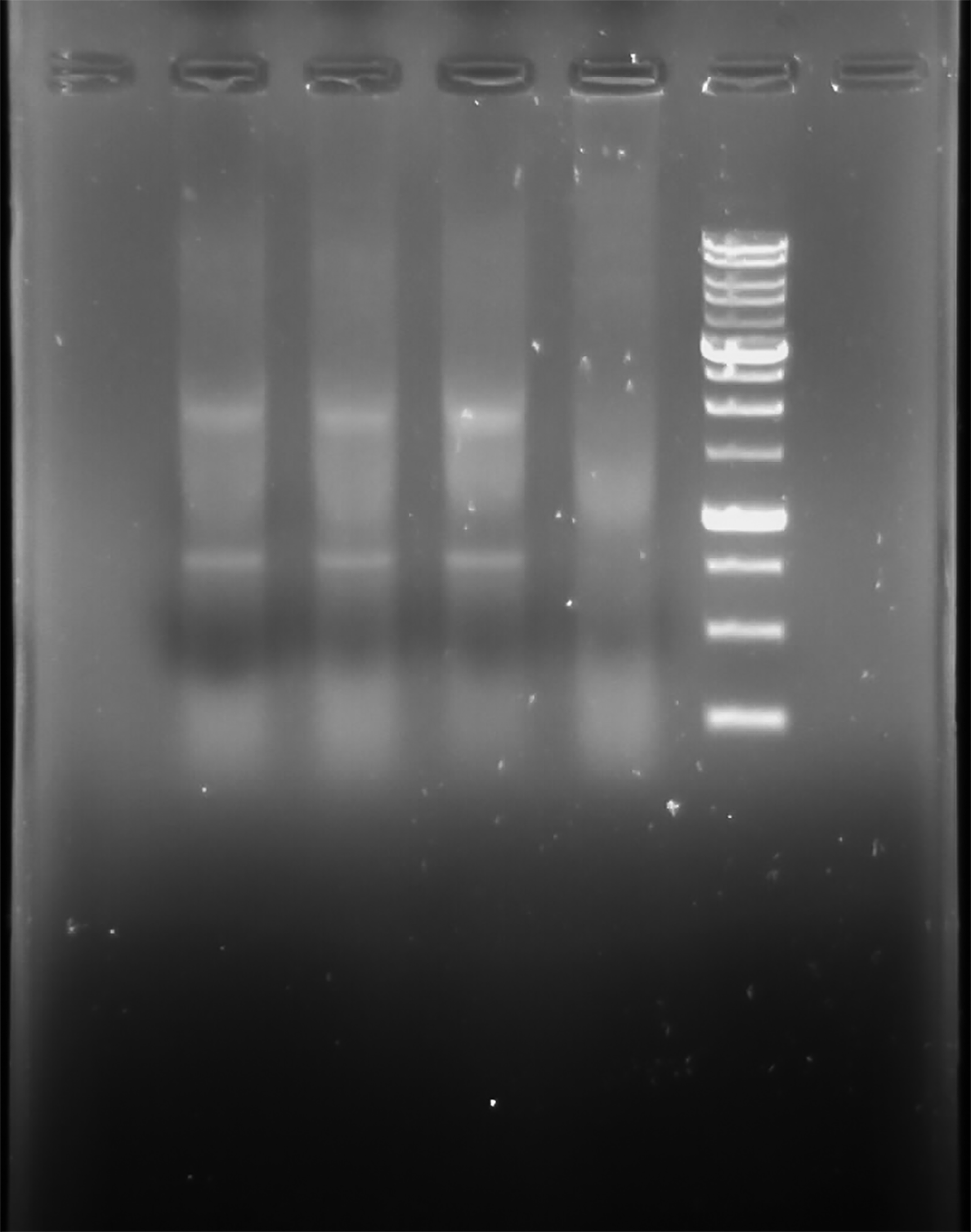

Supplement: S4 Fig — The five right-most lanes were cropped and presented in Fig 5, while the remaining lanes are not related to this study. (TIF) [file pone.0349088.s013.tif]

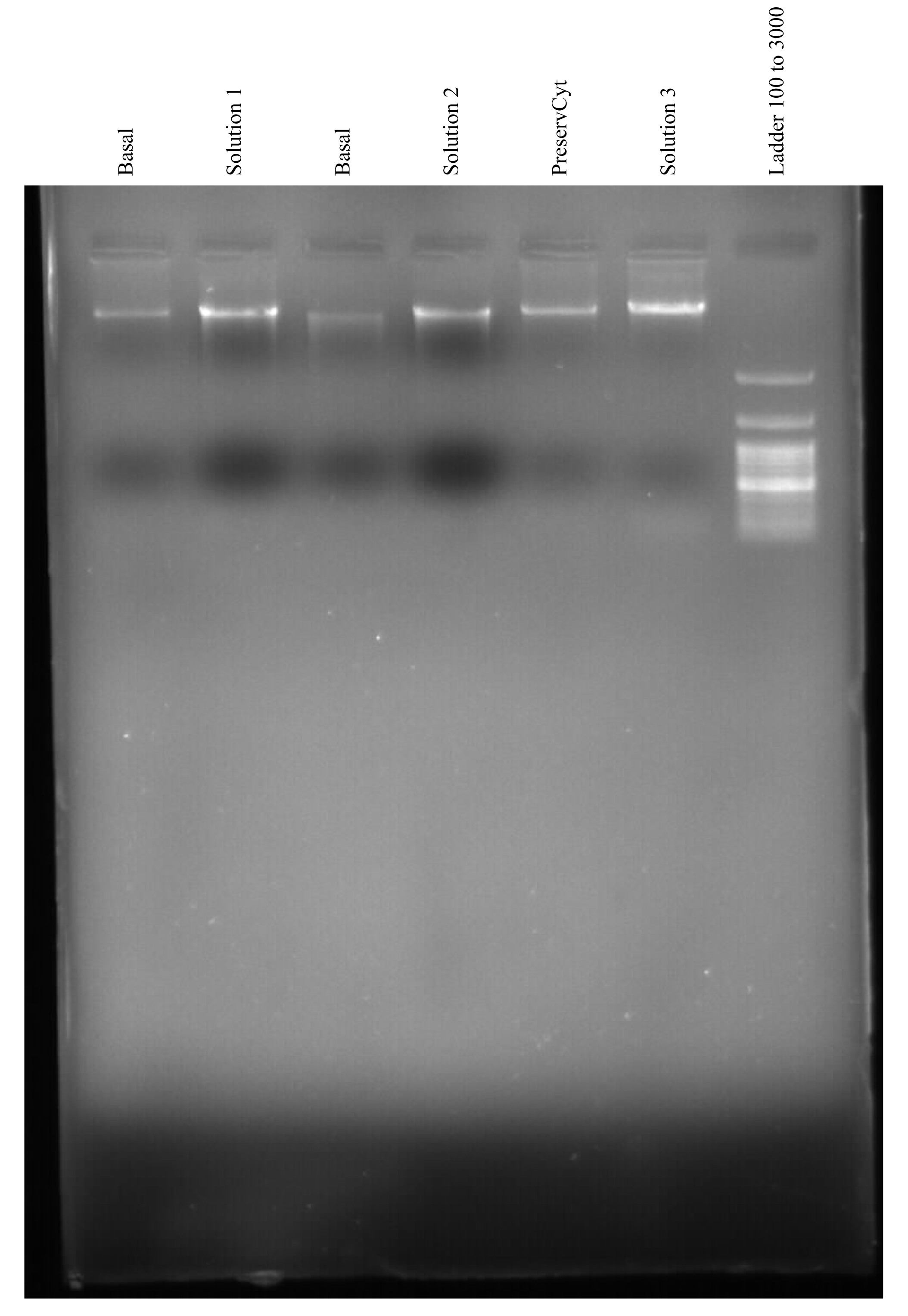

Supplement: S5 Fig — The top row was cropped and presented in Fig 5, while the bottom row shows the E6–E7 oncogenes of HPV, yielding a product size of 906 bp for the experimental runs within the design space. (TIF) [file pone.0349088.s014.tif]

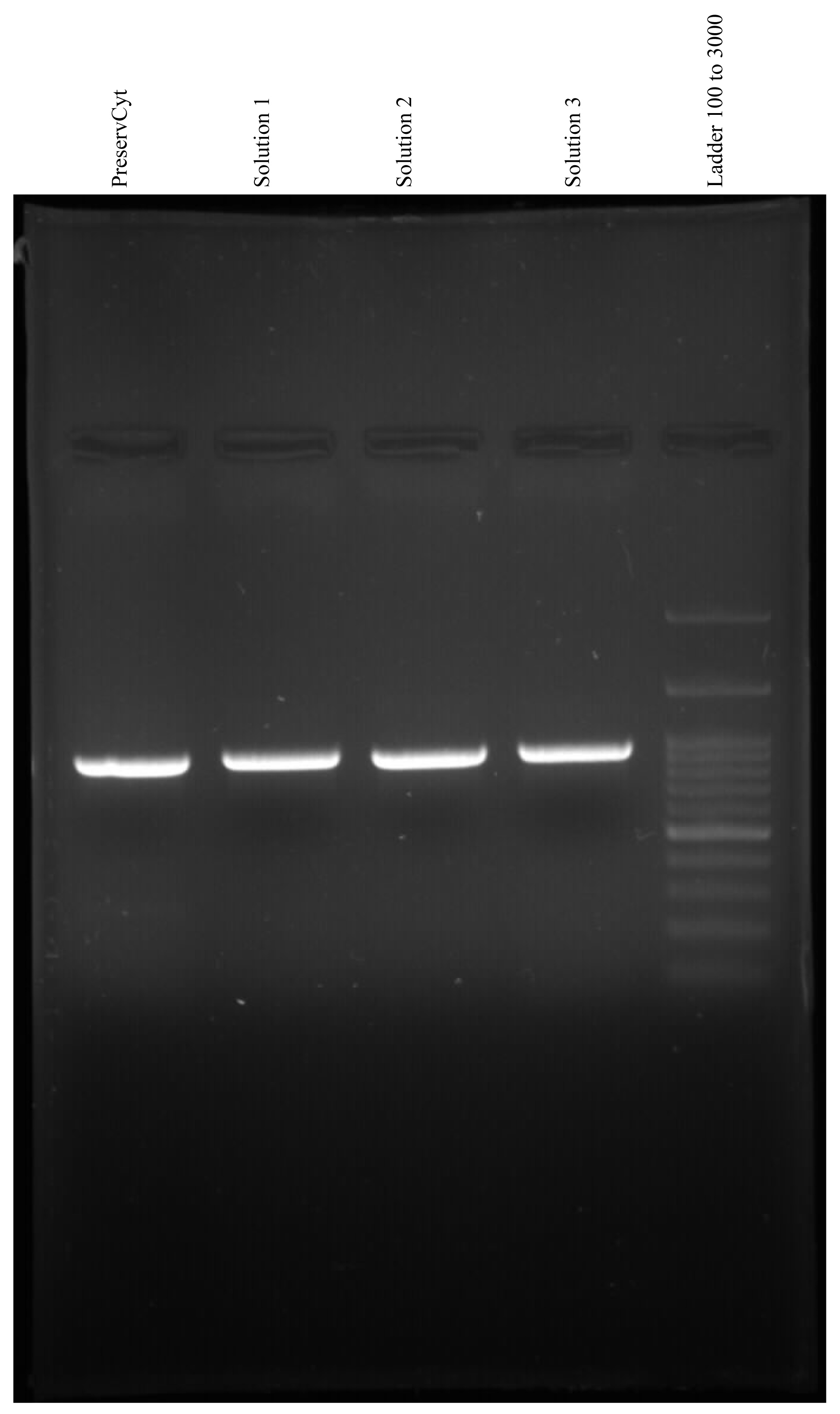

Supplement: S6 Fig — (TIF) [file pone.0349088.s015.tif]

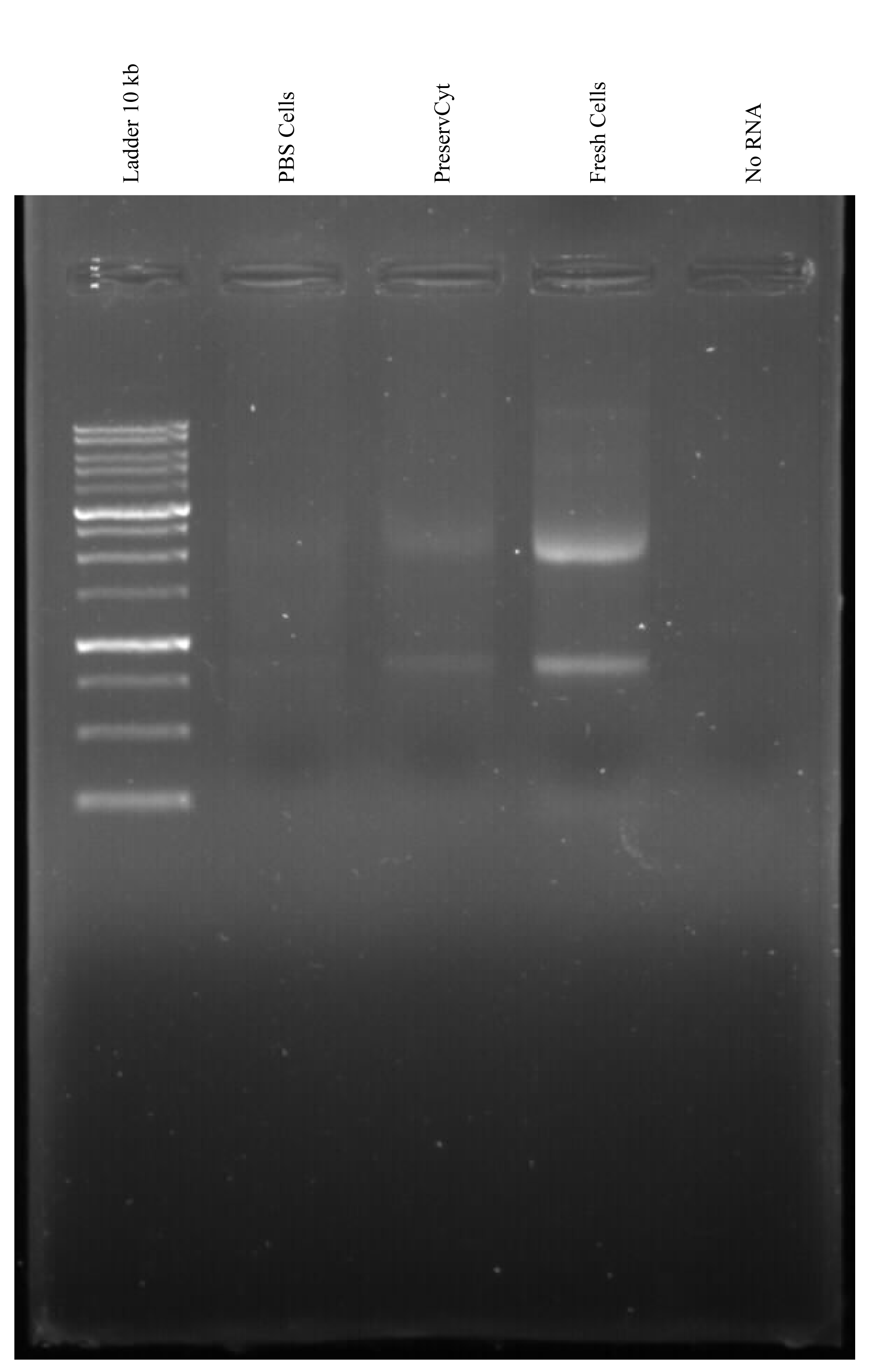

Supplement: S7 Fig — Genomic DNA bands with different intensity after 7 days of storage at room temperature. 1- basal solution with no polyol compounds, Score: + 1, 2- Solution 1, Score: + 2, 3- Replication of basal solution, Score: 0, 4- Solution 2, Score: + 2, 5- PreservCyt, Score: + 1, 6- Solution 3, Score: + 2. (TIF) [file pone.0349088.s016.tif]

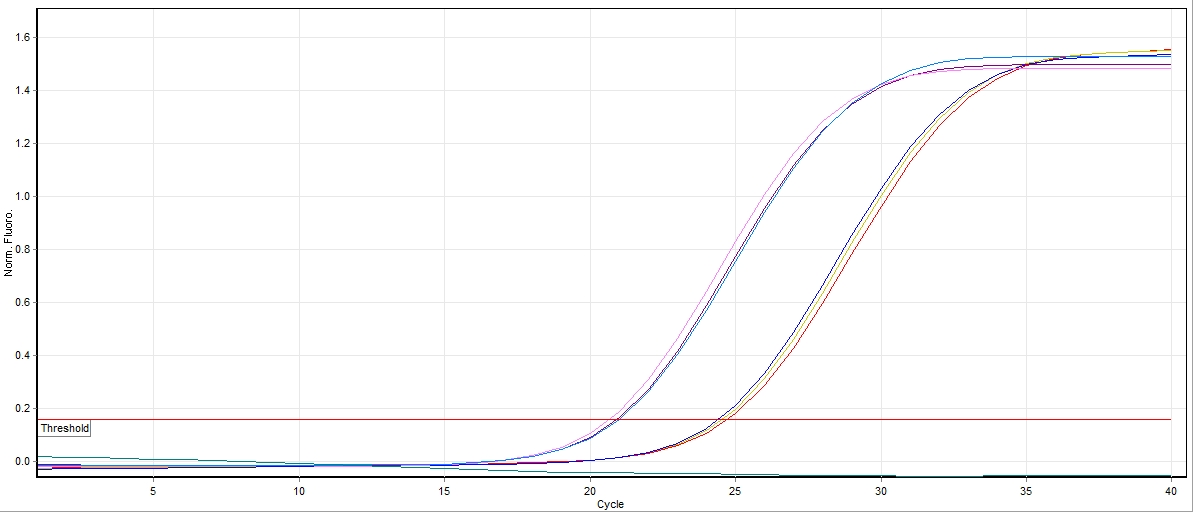

Supplement: S10 Fig — (TIFF) [file pone.0349088.s008.tiff]

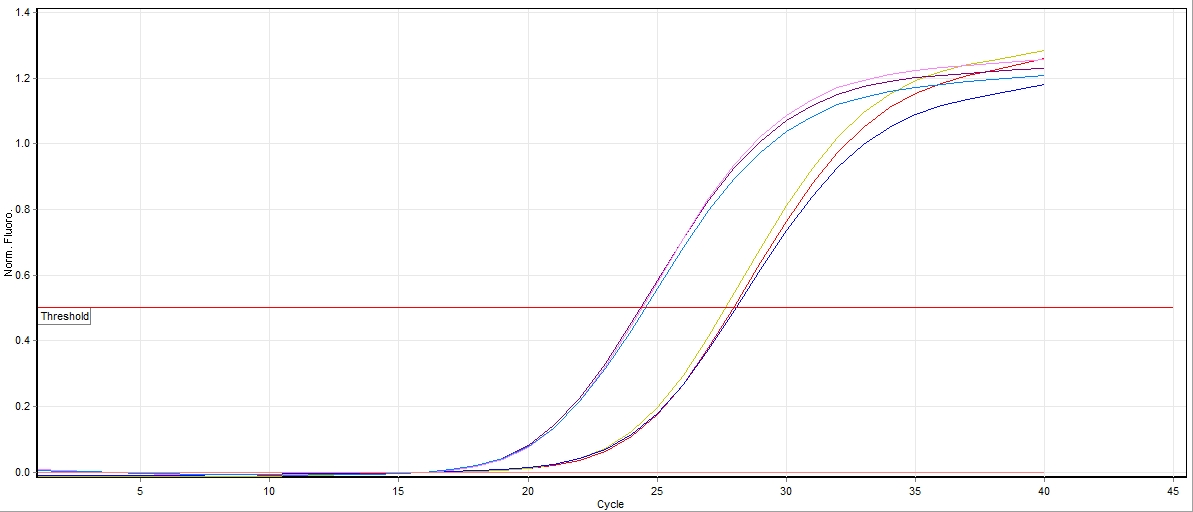

Supplement: S11 Fig — (TIFF) [file pone.0349088.s009.tiff]
