# Supplementary material for: Optimizing an ethanol-based fixative for enhanced nucleic acid preservation in cervical samples using a central composite design approach
Source: PLoS One. 2026 Jun 26;21(6):e0349088. doi: 10.1371/journal.pone.0349088 (PMC13308814; doi:10.1371/journal.pone.0349088)
Supplement: S2 File — (PDF) [file pone.0349088.s006.pdf]

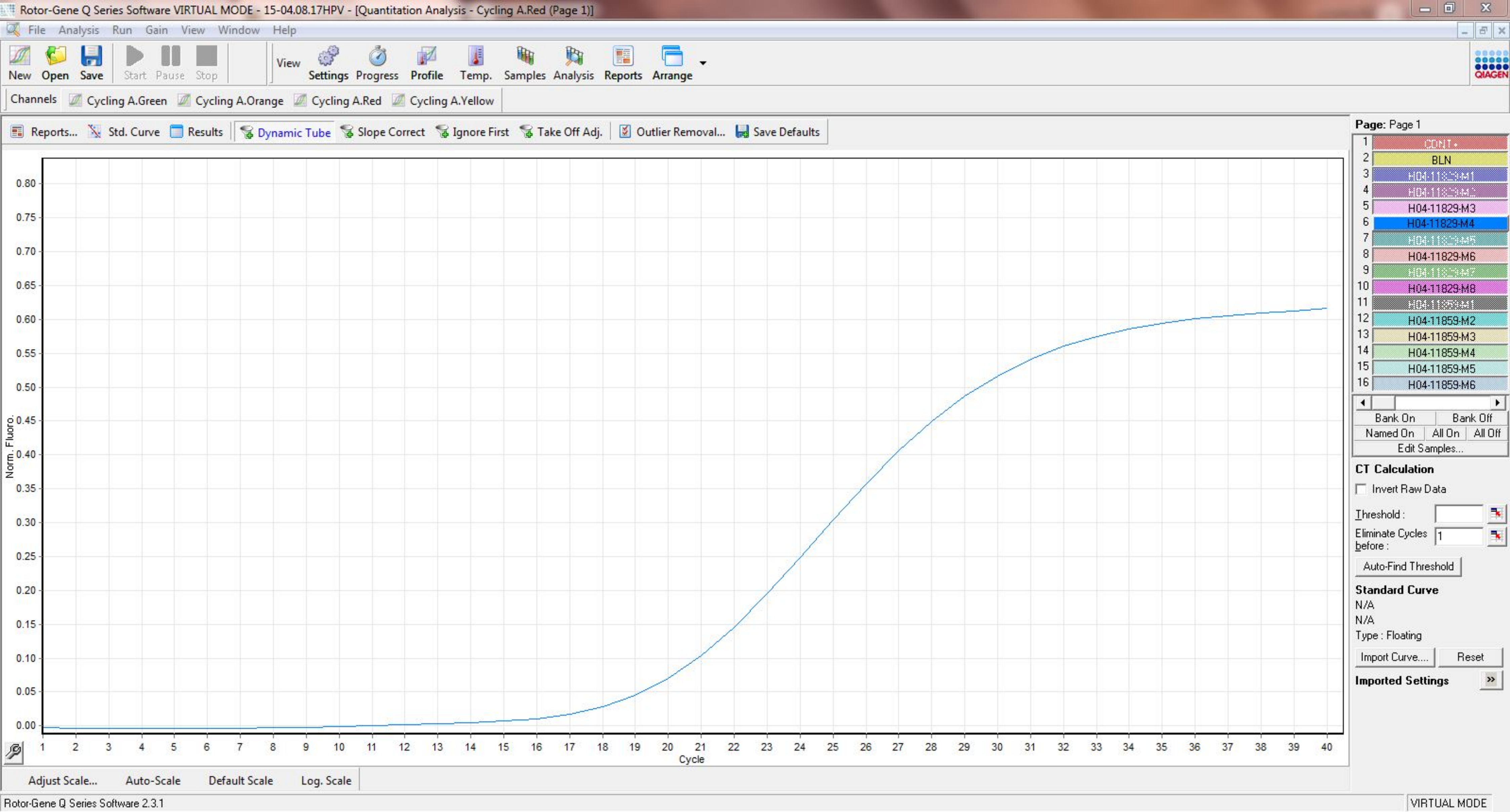

Rotor-Gene Q Series Software VIRTUAL MODE - 15-04.08.17HPV - [Quantitation Analysis - Cycling A.Red (Page 1)]
File Analysis Run Gain View Window Help
New Open Save Start Pause Stop View Settings Progress Profile Temp. Samples Analysis Reports Arrange
QIAGEN
Channels Cycling A.Green Cycling A.Orange Cycling A.Red Cycling A.Yellow
Reports... Std. Curve Results Dynamic Tube Slope Correct Ignore First Take Off Adj. Outlier Removal... Save Defaults
Norm. Fluoro.
0.80 0.75 0.70 0.65 0.60 0.55 0.50 0.45 0.40 0.35 0.30 0.25 0.20 0.15 0.10 0.05 0.00
1 2 3 4 5 6 7 8 9 10 11 12 13 14 15 16 17 18 19 20 21 22 23 24 25 26 27 28 29 30 31 32 33 34 35 36 37 38 39 40
Cycle
Page: Page 1
1 CONT+
2 BLN
5 H04-11829-M3
6 H04-11829-M4
8 H04-11829-M6
10 H04-11829-M8
12 H04-11859-M2
13 H04-11859-M3
14 H04-11859-M4
15 H04-11859-M5
16 H04-11859-M6
Bank On Bank Off
Named On All On All Off
Edit Samples...
CT Calculation
Invert Raw Data
Threshold :
Eliminate Cycles before : 1
Auto-Find Threshold
Standard Curve
N/A
N/A
Type : Floating
Import Curve... Reset
Imported Settings
Adjust Scale... Auto-Scale Default Scale Log. Scale
Rotor-Gene Q Series Software 2.3.1
VIRTUAL MODE

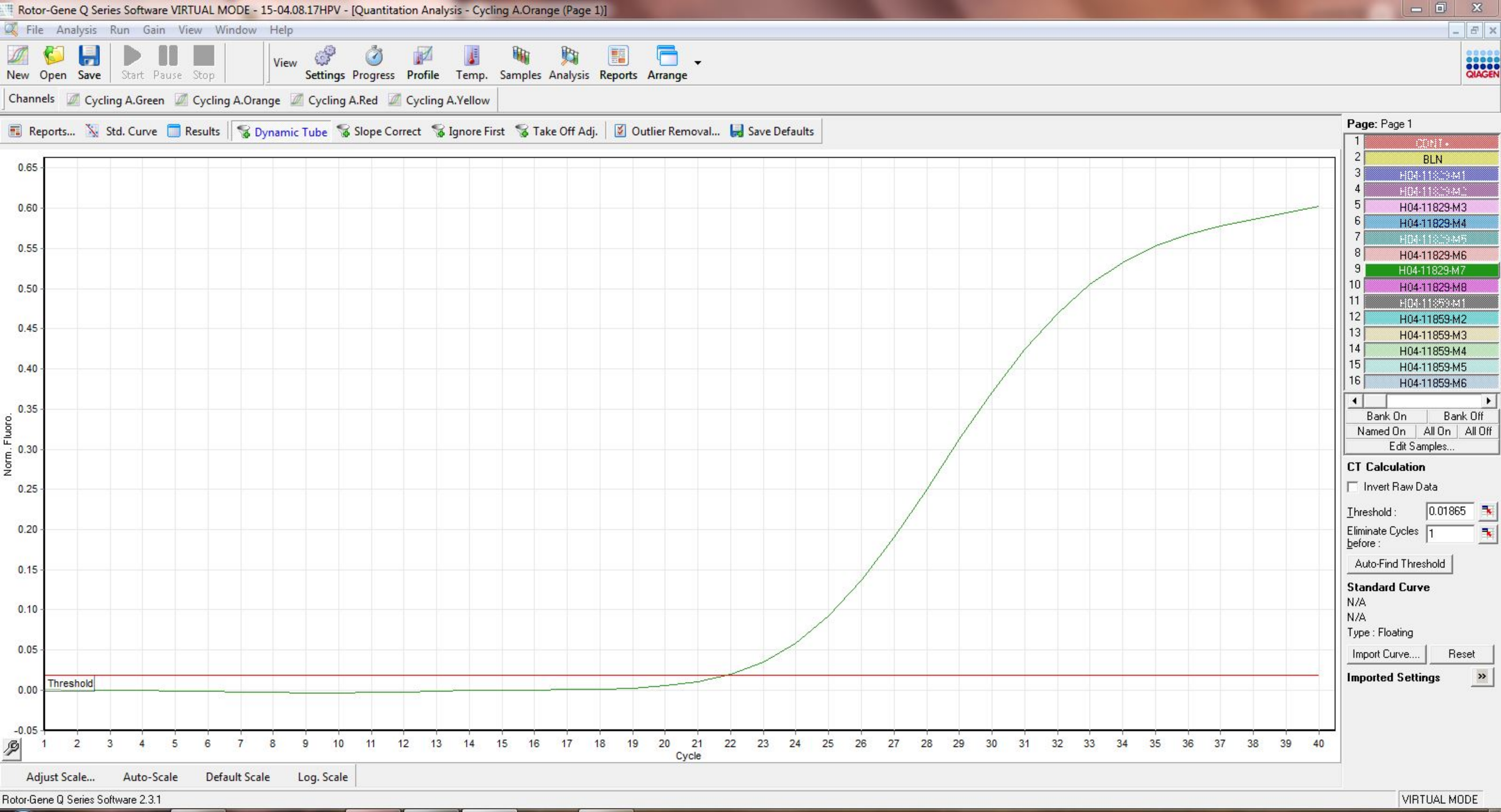
Rotor-Gene Q Series Software VIRTUAL MODE - 15-04.08.17HPV - [Quantitation Analysis - Cycling A.Orange (Page 1)]
File Analysis Run Gain View Window Help
New Open Save Start Pause Stop View Settings Progress Profile Temp. Samples Analysis Reports Arrange
QIAGEN
Channels Cycling A.Green Cycling A.Orange Cycling A.Red Cycling A.Yellow
Reports... Std. Curve Results Dynamic Tube Slope Correct Ignore First Take Off Adj. Outlier Removal... Save Defaults
Norm. Fluoro.
Cycle
Threshold
Page: Page 1
2 BLN
5 H04-11829-M3
6 H04-11829-M4
8 H04-11829-M6
9 H04-11829-M7
10 H04-11829-M8
12 H04-11859-M2
13 H04-11859-M3
14 H04-11859-M4
15 H04-11859-M5
16 H04-11859-M6
Bank On Bank Off
Named On All On All Off
Edit Samples...
CT Calculation
Invert Raw Data
Threshold : 0.01865
Eliminate Cycles before : 1
Auto-Find Threshold
Standard Curve
N/A
N/A
Type : Floating
Import Curve.... Reset
Imported Settings
Adjust Scale... Auto-Scale Default Scale Log. Scale
Rotor-Gene Q Series Software 2.3.1
VIRTUAL MODE

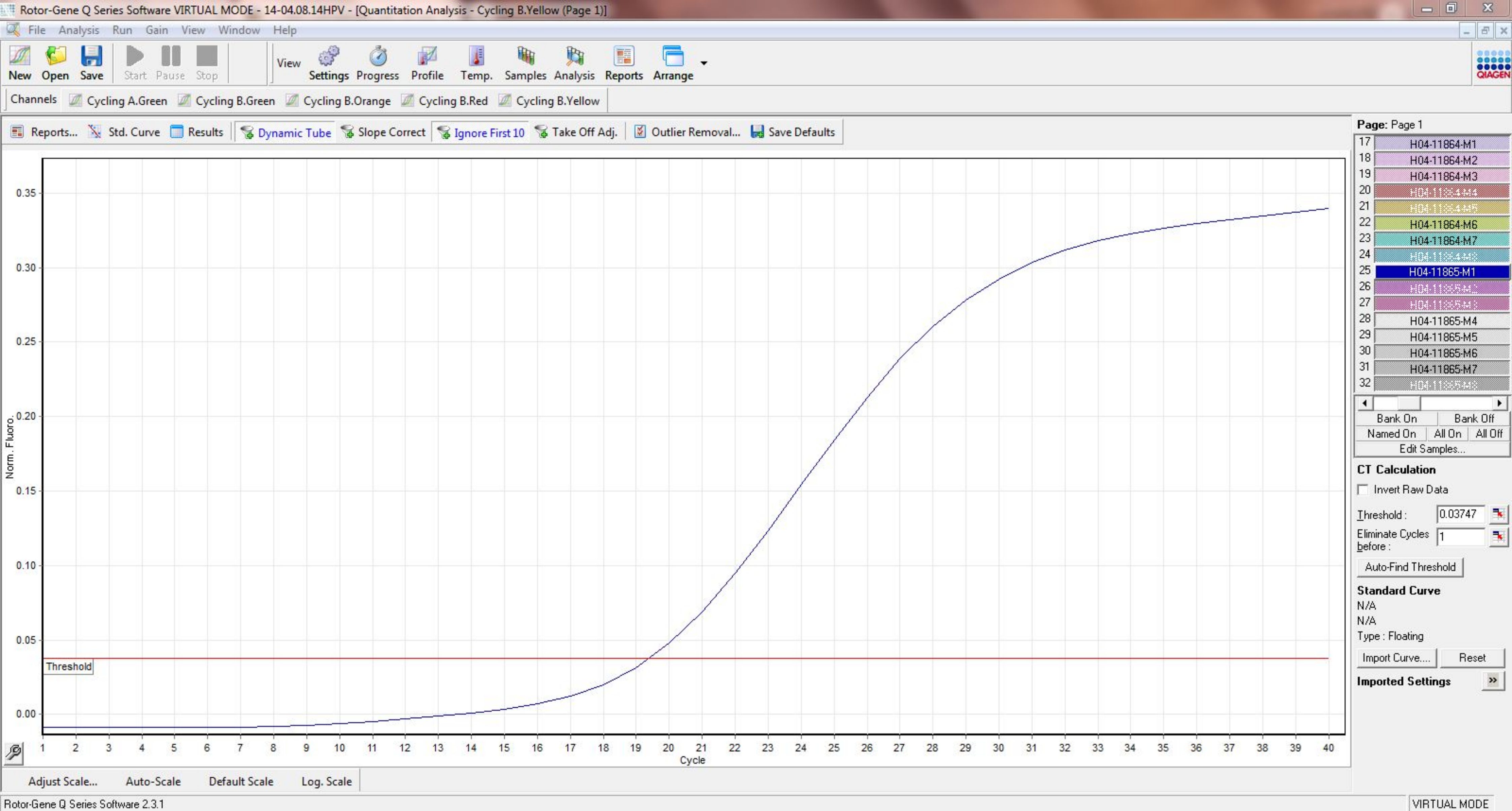
Rotor-Gene Q Series Software VIRTUAL MODE - 14-04.08.14HPV - [Quantitation Analysis - Cycling B.Yellow (Page 1)]
File Analysis Run Gain View Window Help
New Open Save Start Pause Stop View Settings Progress Profile Temp. Samples Analysis Reports Arrange
QIAGEN
Channels Cycling A.Green Cycling B.Green Cycling B.Orange Cycling B.Red Cycling B.Yellow
Reports... Std. Curve Results Dynamic Tube Slope Correct Ignore First 10 Take Off Adj. Outlier Removal... Save Defaults
Norm. Fluoro.
0.35
0.30
0.25
0.20
0.15
0.10
0.05
0.00
Threshold
1 2 3 4 5 6 7 8 9 10 11 12 13 14 15 16 17 18 19 20 21 22 23 24 25 26 27 28 29 30 31 32 33 34 35 36 37 38 39 40
Cycle
Page: Page 1
17 H04-11864-M1
18 H04-11864-M2
19 H04-11864-M3
20
21
22 H04-11864-M6
23 H04-11864-M7
24
25 H04-11865-M1
26
27
28 H04-11865-M4
29 H04-11865-M5
30 H04-11865-M6
31 H04-11865-M7
32
Bank On Bank Off
Named On All On All Off
Edit Samples...
CT Calculation
Invert Raw Data
Threshold : 0.03747
Eliminate Cycles before : 1
Auto-Find Threshold
Standard Curve
N/A
N/A
Type : Floating
Import Curve... Reset
Imported Settings
Adjust Scale... Auto-Scale Default Scale Log. Scale
Rotor-Gene Q Series Software 2.3.1
VIRTUAL MODE

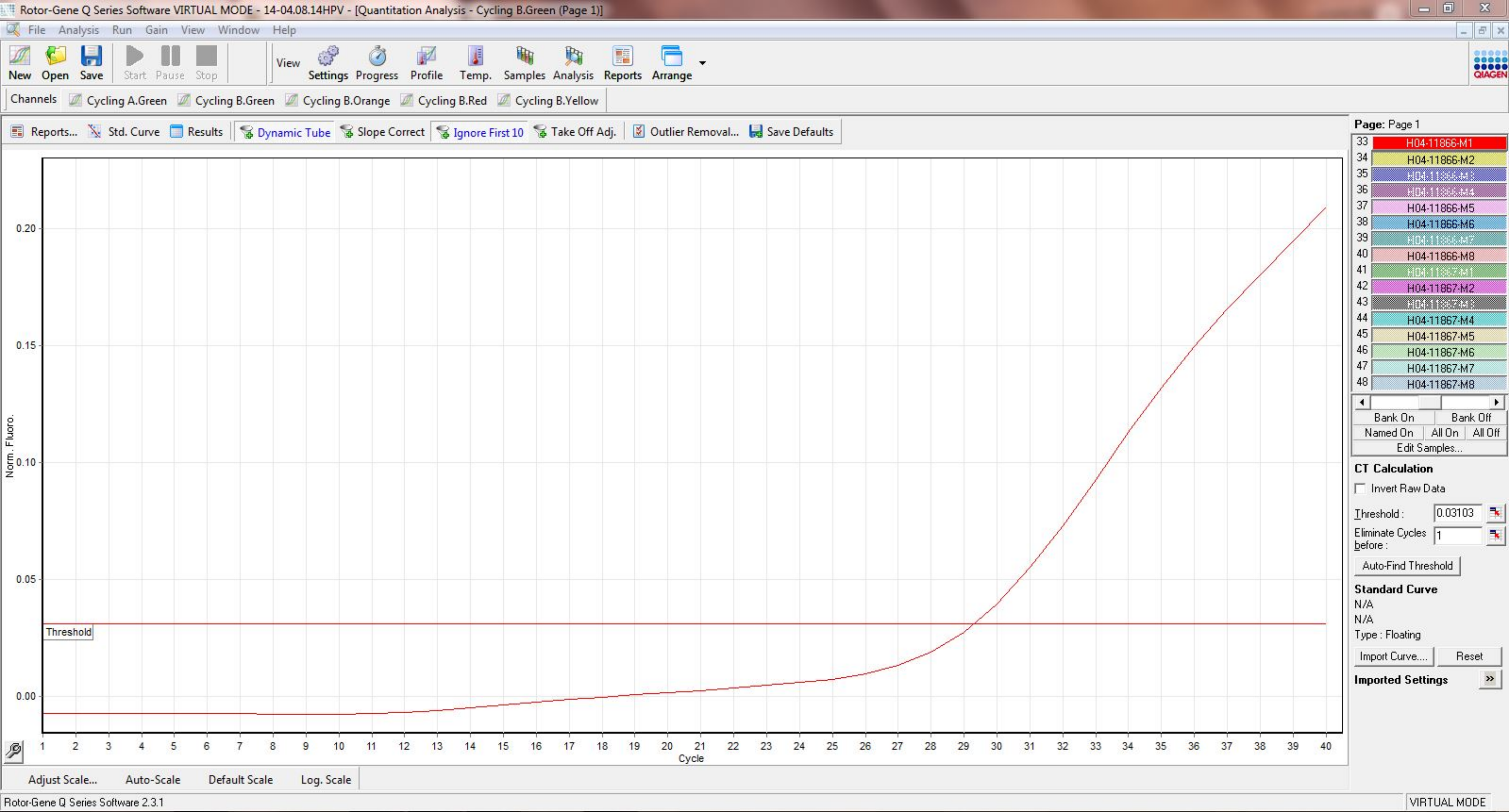
Rotor-Gene Q Series Software VIRTUAL MODE - 14-04.08.14HPV - [Quantitation Analysis - Cycling B.Green (Page 1)]
File Analysis Run Gain View Window Help
New Open Save Start Pause Stop View Settings Progress Profile Temp. Samples Analysis Reports Arrange
QIAGEN
Channels Cycling A.Green Cycling B.Green Cycling B.Orange Cycling B.Red Cycling B.Yellow
Reports... Std. Curve Results Dynamic Tube Slope Correct Ignore First 10 Take Off Adj. Outlier Removal... Save Defaults
Norm. Fluoro.
0.20
0.15
0.10
0.05
0.00
Threshold
1 2 3 4 5 6 7 8 9 10 11 12 13 14 15 16 17 18 19 20 21 22 23 24 25 26 27 28 29 30 31 32 33 34 35 36 37 38 39 40
Cycle
Adjust Scale... Auto-Scale Default Scale Log. Scale
Page: Page 1
33 H04-11866-M1
34 H04-11866-M2
35
36
37 H04-11866-M5
38 H04-11866-M6
39
40 H04-11866-M8
41
42 H04-11867-M2
43
44 H04-11867-M4
45 H04-11867-M5
46 H04-11867-M6
47 H04-11867-M7
48 H04-11867-M8
Bank On Bank Off
Named On All On All Off
Edit Samples...
CT Calculation
Invert Raw Data
Threshold : 0.03103
Eliminate Cycles before : 1
Auto-Find Threshold
Standard Curve
N/A
N/A
Type : Floating
Import Curve... Reset
Imported Settings
Rotor-Gene Q Series Software 2.3.1
VIRTUAL MODE

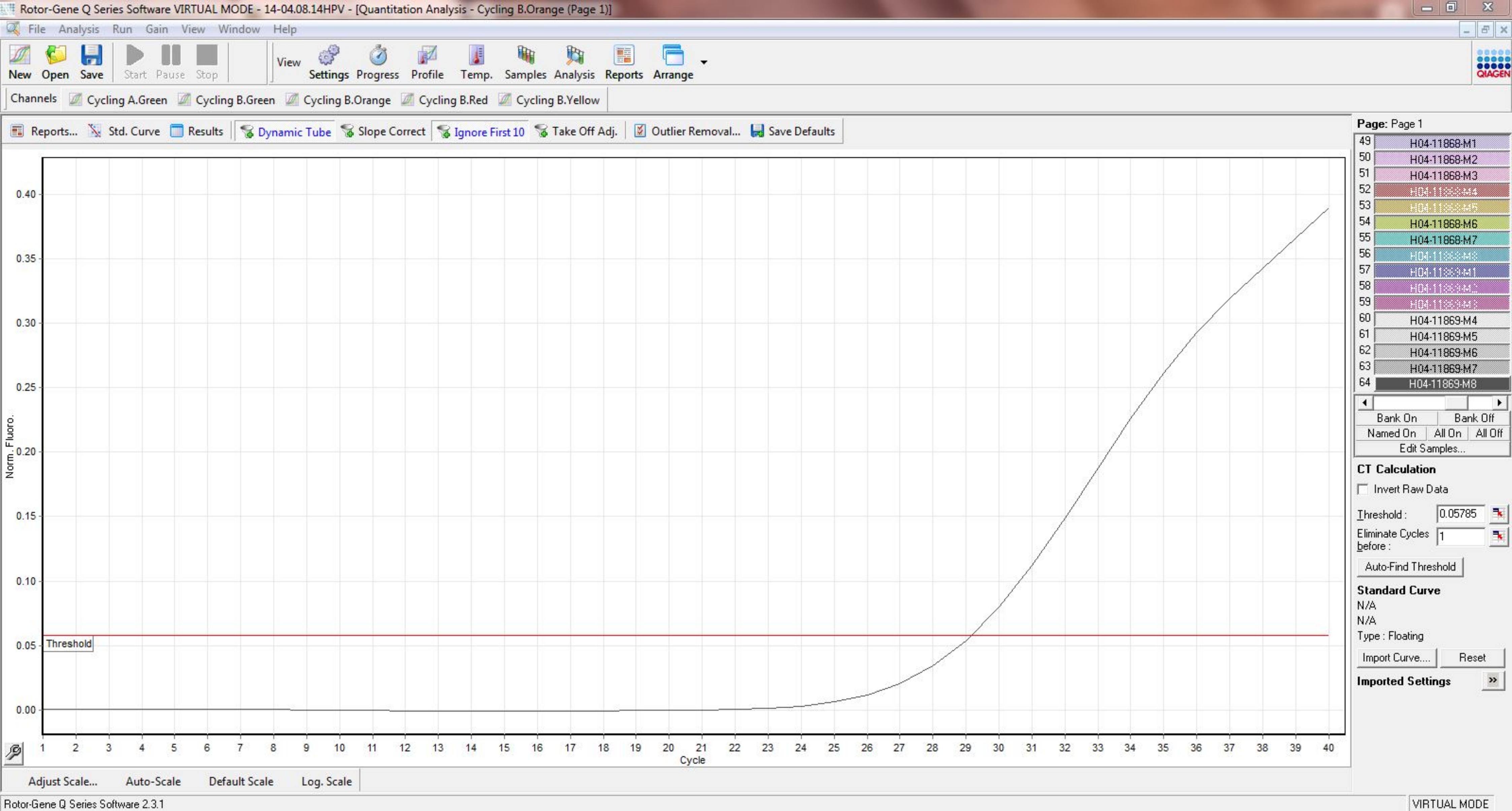
Rotor-Gene Q Series Software VIRTUAL MODE - 14-04.08.14HPV - [Quantitation Analysis - Cycling B.Orange (Page 1)]
File Analysis Run Gain View Window Help
New Open Save Start Pause Stop View Settings Progress Profile Temp. Samples Analysis Reports Arrange
QIAGEN
Channels Cycling A.Green Cycling B.Green Cycling B.Orange Cycling B.Red Cycling B.Yellow
Reports... Std. Curve Results Dynamic Tube Slope Correct Ignore First 10 Take Off Adj. Outlier Removal... Save Defaults
Norm. Fluoro.
0.40
0.35
0.30
0.25
0.20
0.15
0.10
0.05
0.00
Threshold
1 2 3 4 5 6 7 8 9 10 11 12 13 14 15 16 17 18 19 20 21 22 23 24 25 26 27 28 29 30 31 32 33 34 35 36 37 38 39 40
Cycle
Adjust Scale... Auto-Scale Default Scale Log. Scale
Page: Page 1
49 H04-11868-M1
50 H04-11868-M2
51 H04-11868-M3
52
53
54 H04-11868-M6
55 H04-11868-M7
56
57
58
59
60 H04-11869-M4
61 H04-11869-M5
62 H04-11869-M6
63 H04-11869-M7
64 H04-11869-M8
Bank On Bank Off
Named On All On All Off
Edit Samples...
CT Calculation
Invert Raw Data
Threshold : 0.05785
Eliminate Cycles before : 1
Auto-Find Threshold
Standard Curve
N/A
N/A
Type : Floating
Import Curve... Reset
Imported Settings
Rotor-Gene Q Series Software 2.3.1
VIRTUAL MODE

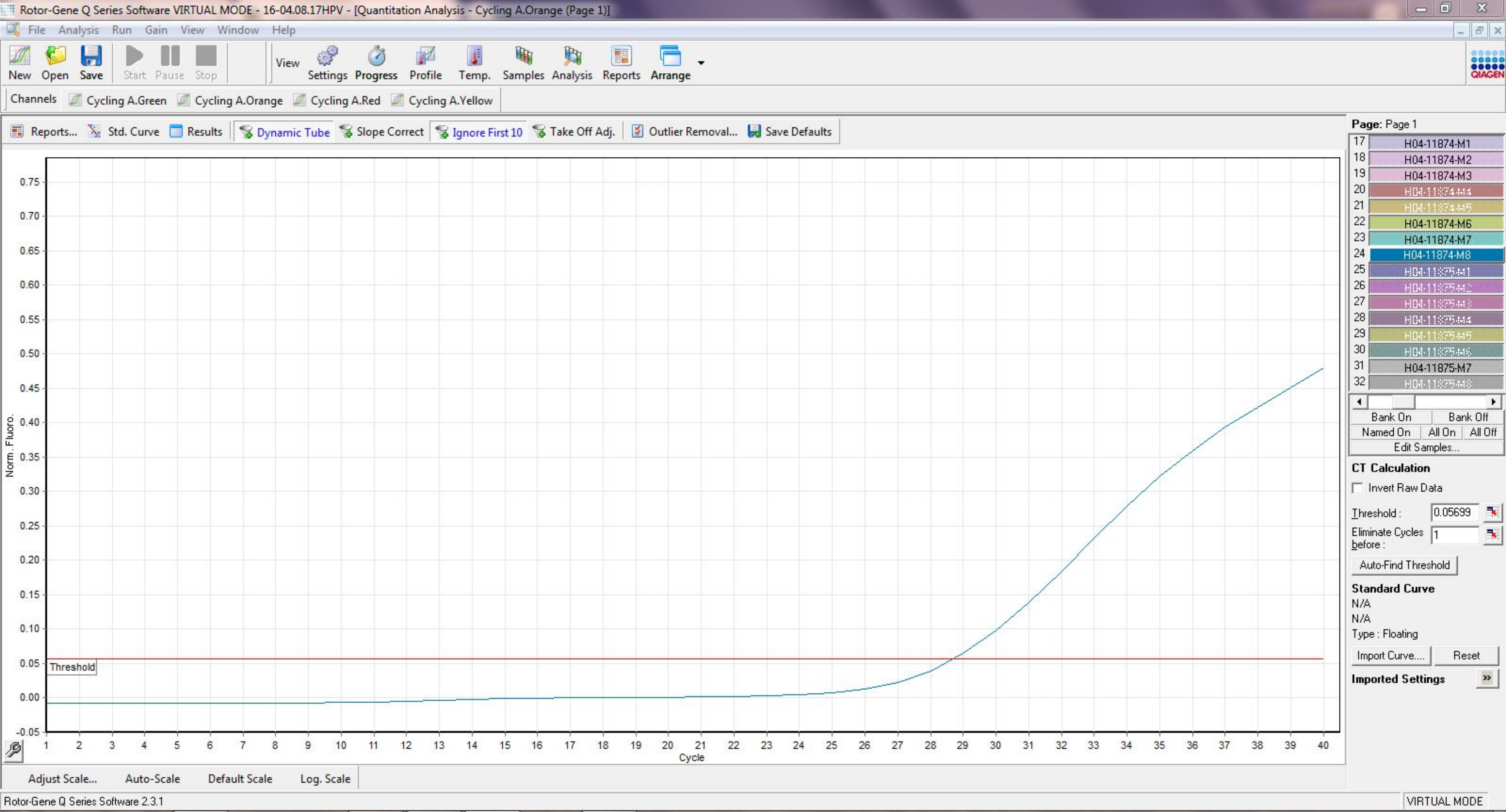
Rotor-Gene Q Series Software VIRTUAL MODE - 16-04.08.17HPV - [Quantitation Analysis - Cycling A.Orange (Page 1)]
File Analysis Run Gain View Window Help
New Open Save Start Pause Stop View Settings Progress Profile Temp. Samples Analysis Reports Arrange
QIAGEN
Channels Cycling A.Green Cycling A.Orange Cycling A.Red Cycling A.Yellow
Reports... Std. Curve Results Dynamic Tube Slope Correct Ignore First 10 Take Off Adj. Outlier Removal... Save Defaults
Norm. Fluoro.
Threshold
Cycle
Page: Page 1
17 H04-11874-M1
18 H04-11874-M2
19 H04-11874-M3
20
21
22 H04-11874-M6
23 H04-11874-M7
24 H04-11874-M8
25
26
27
28
29
30
31 H04-11875-M7
32
Bank On Bank Off
Named On All On All Off
Edit Samples...
CT Calculation
Invert Raw Data
Threshold : 0.05699
Eliminate Cycles before : 1
Auto-Find Threshold
Standard Curve
N/A
N/A
Type : Floating
Import Curve... Reset
Imported Settings
Adjust Scale... Auto-Scale Default Scale Log. Scale
Rotor-Gene Q Series Software 2.3.1
VIRTUAL MODE

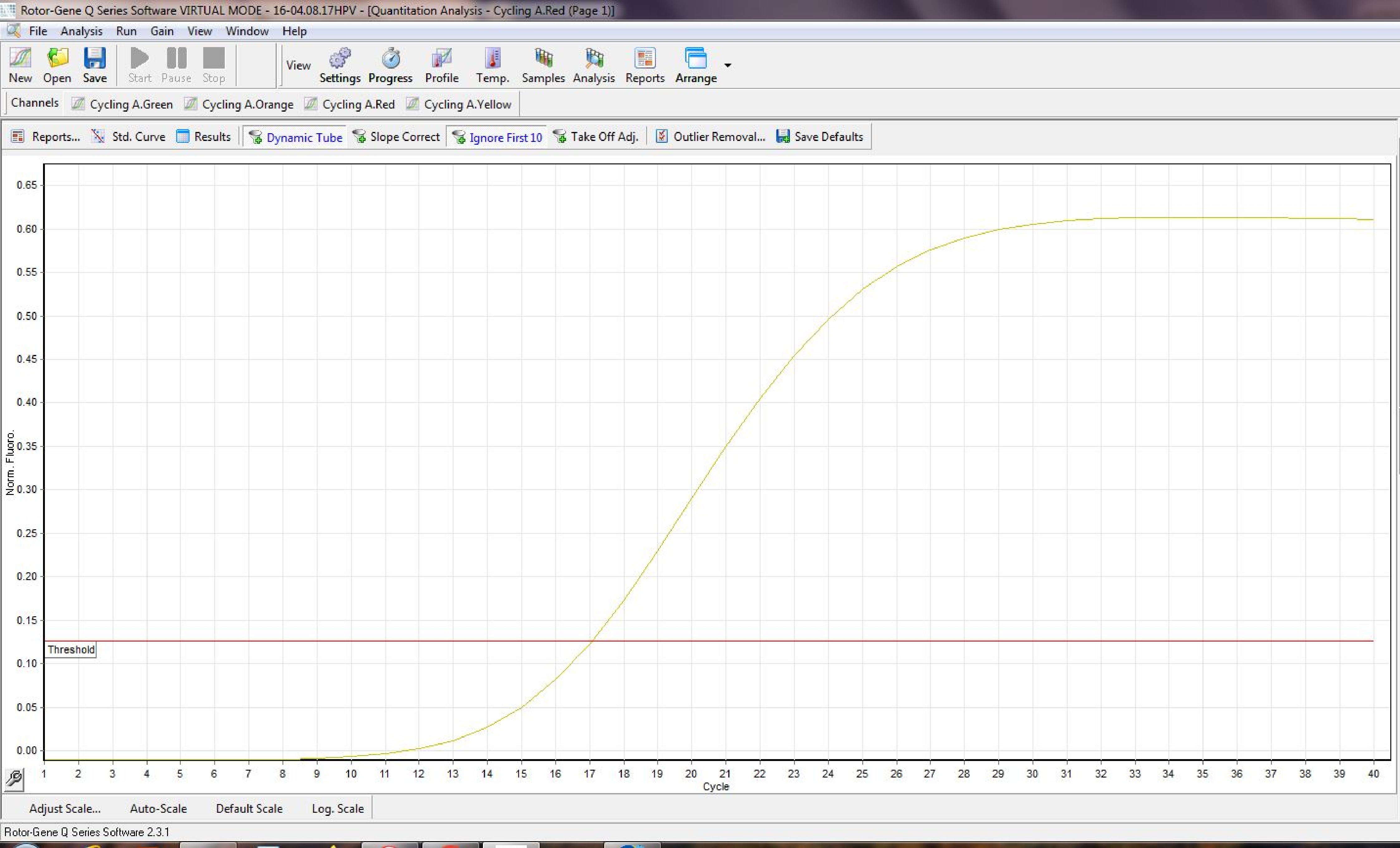
Rotor-Gene Q Series Software VIRTUAL MODE - 16-04.08.17HPV - [Quantitation Analysis - Cycling A.Red (Page 1)]
File Analysis Run Gain View Window Help
New Open Save Start Pause Stop View Settings Progress Profile Temp. Samples Analysis Reports Arrange
Channels Cycling A.Green Cycling A.Orange Cycling A.Red Cycling A.Yellow
Reports... Std. Curve Results Dynamic Tube Slope Correct Ignore First 10 Take Off Adj. Outlier Removal... Save Defaults
Norm. Fluoro.
0.65
0.60
0.55
0.50
0.45
0.40
0.35
0.30
0.25
0.20
0.15
0.10
0.05
0.00
Threshold
1 2 3 4 5 6 7 8 9 10 11 12 13 14 15 16 17 18 19 20 21 22 23 24 25 26 27 28 29 30 31 32 33 34 35 36 37 38 39 40
Cycle
Adjust Scale... Auto-Scale Default Scale Log. Scale
Rotor-Gene Q Series Software 2.3.1

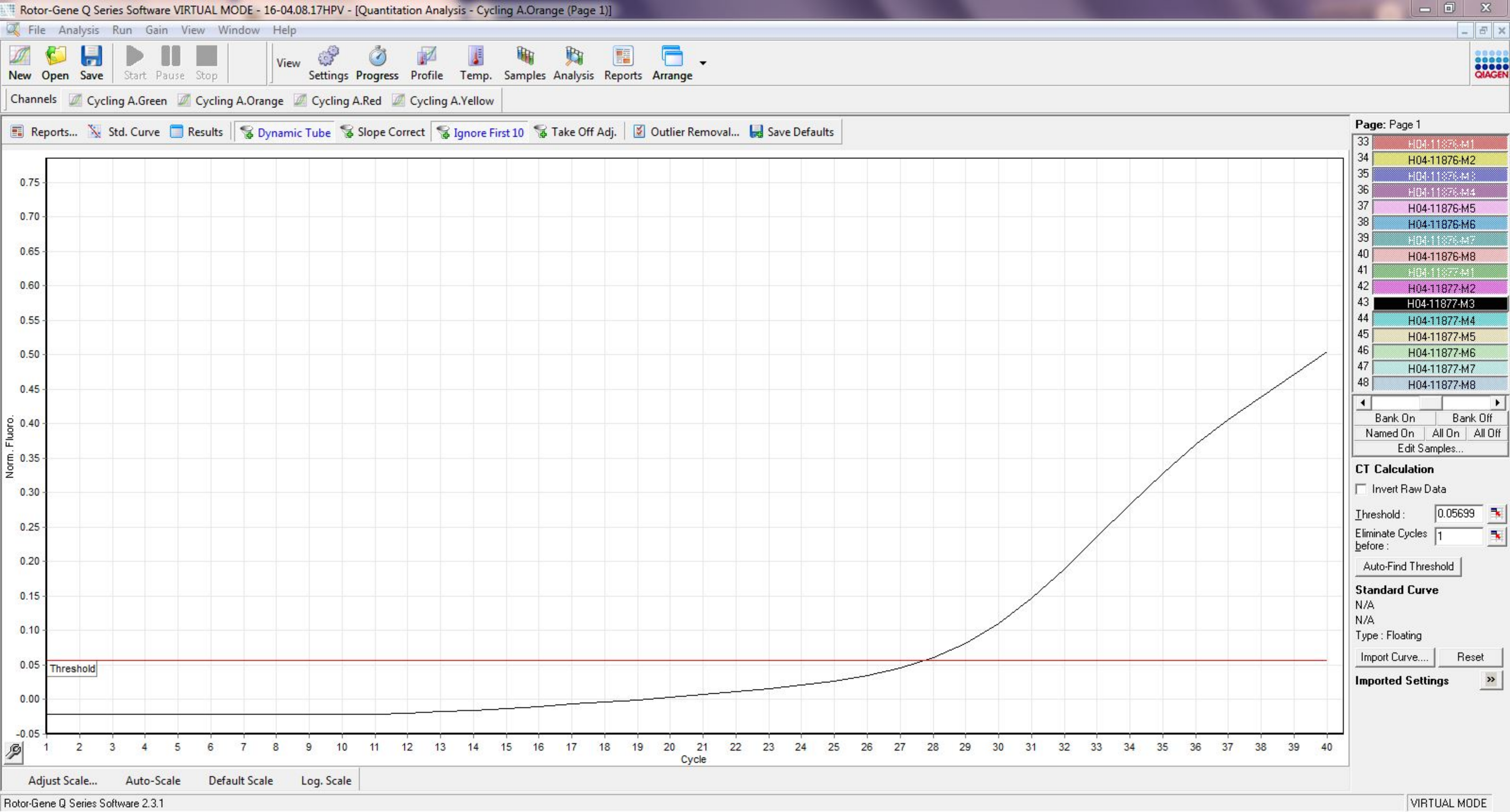
Rotor-Gene Q Series Software VIRTUAL MODE - 16-04.08.17HPV - [Quantitation Analysis - Cycling A.Orange (Page 1)]
File Analysis Run Gain View Window Help
New Open Save Start Pause Stop View Settings Progress Profile Temp. Samples Analysis Reports Arrange
QIAGEN
Channels Cycling A.Green Cycling A.Orange Cycling A.Red Cycling A.Yellow
Reports... Std. Curve Results Dynamic Tube Slope Correct Ignore First 10 Take Off Adj. Outlier Removal... Save Defaults
Norm. Fluoro.
0.75 0.70 0.65 0.60 0.55 0.50 0.45 0.40 0.35 0.30 0.25 0.20 0.15 0.10 0.05 0.00 -0.05
Threshold
1 2 3 4 5 6 7 8 9 10 11 12 13 14 15 16 17 18 19 20 21 22 23 24 25 26 27 28 29 30 31 32 33 34 35 36 37 38 39 40
Cycle
Adjust Scale... Auto-Scale Default Scale Log. Scale
Page: Page 1
33
34 H04-11876-M2
35
36
37 H04-11876-M5
38 H04-11876-M6
39
40 H04-11876-M8
41
42 H04-11877-M2
43 H04-11877-M3
44 H04-11877-M4
45 H04-11877-M5
46 H04-11877-M6
47 H04-11877-M7
48 H04-11877-M8
Bank On Bank Off
Named On All On All Off
Edit Samples...
CT Calculation
Invert Raw Data
Threshold : 0.05699
Eliminate Cycles before : 1
Auto-Find Threshold
Standard Curve
N/A
N/A
Type : Floating
Import Curve... Reset
Imported Settings
Rotor-Gene Q Series Software 2.3.1
VIRTUAL MODE

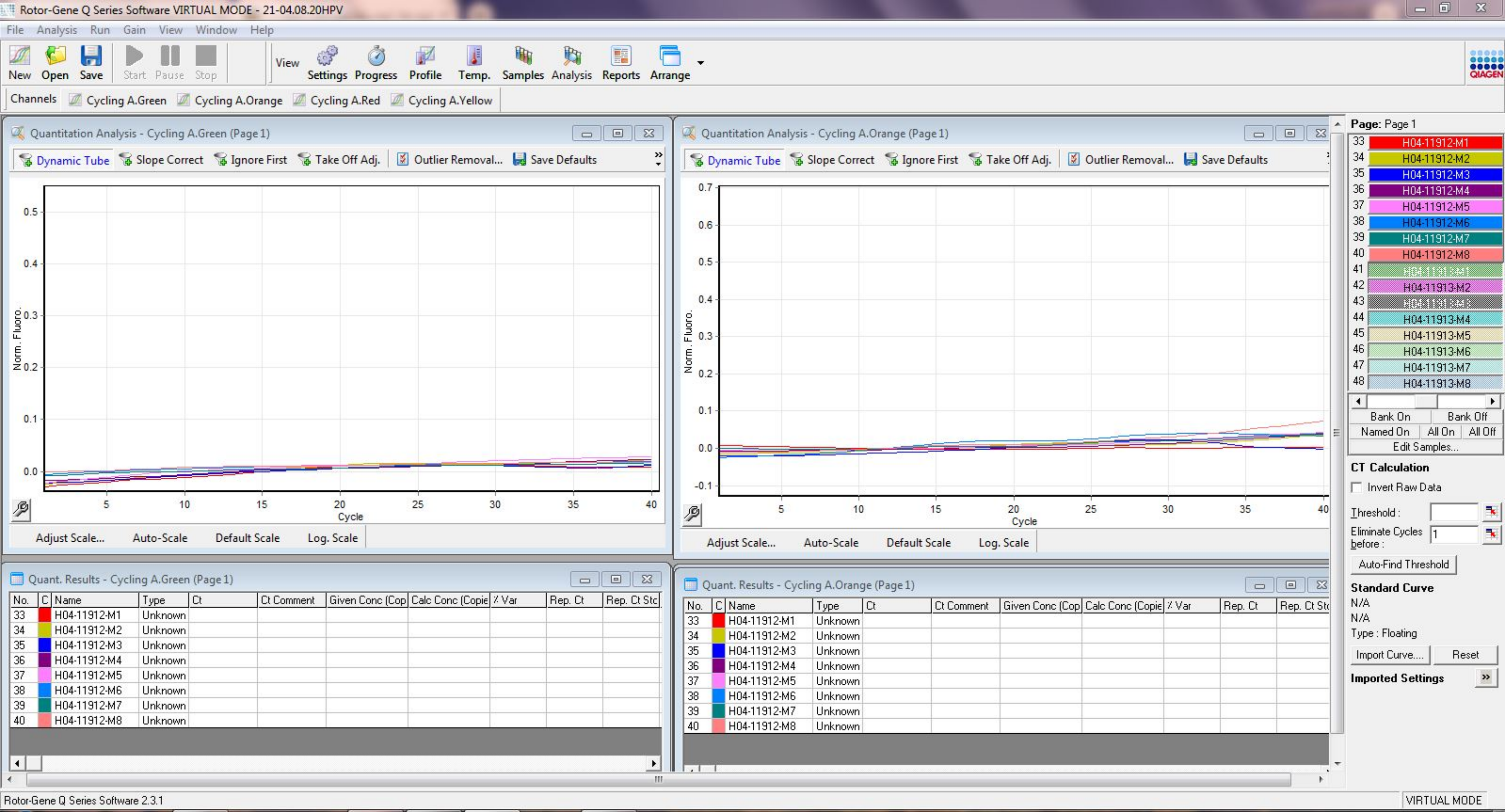
Rotor-Gene Q Series Software VIRTUAL MODE - 21-04.08.20HPV
File Analysis Run Gain View Window Help
New Open Save Start Pause Stop View Settings Progress Profile Temp. Samples Analysis Reports Arrange
QIAGEN
Channels Cycling A.Green Cycling A.Orange Cycling A.Red Cycling A.Yellow
Quantitation Analysis - Cycling A.Green (Page 1)
Dynamic Tube Slope Correct Ignore First Take Off Adj. Outlier Removal... Save Defaults
Norm. Fluoro.
Cycle
Adjust Scale... Auto-Scale Default Scale Log. Scale
Quantitation Analysis - Cycling A.Orange (Page 1)
Dynamic Tube Slope Correct Ignore First Take Off Adj. Outlier Removal... Save Defaults
Norm. Fluoro.
Cycle
Adjust Scale... Auto-Scale Default Scale Log. Scale
Page: Page 1
33 H04-11912-M1
34 H04-11912-M2
35 H04-11912-M3
36 H04-11912-M4
37 H04-11912-M5
38 H04-11912-M6
39 H04-11912-M7
40 H04-11912-M8
42 H04-11913-M2
44 H04-11913-M4
45 H04-11913-M5
46 H04-11913-M6
47 H04-11913-M7
48 H04-11913-M8
Bank On Bank Off
Named On All On All Off
Edit Samples...
CT Calculation
Invert Raw Data
Threshold :
Eliminate Cycles before : 1
Auto-Find Threshold
Standard Curve
N/A
N/A
Type : Floating
Import Curve... Reset
Imported Settings
Quant. Results - Cycling A.Green (Page 1)
No. C Name Type Ct Ct Comment Given Conc (Cop Calc Conc (Copie % Var Rep. Ct Rep. Ct Std
33 H04-11912-M1 Unknown
34 H04-11912-M2 Unknown
35 H04-11912-M3 Unknown
36 H04-11912-M4 Unknown
37 H04-11912-M5 Unknown
38 H04-11912-M6 Unknown
39 H04-11912-M7 Unknown
40 H04-11912-M8 Unknown
Quant. Results - Cycling A.Orange (Page 1)
No. C Name Type Ct Ct Comment Given Conc (Cop Calc Conc (Copie % Var Rep. Ct Rep. Ct Std
33 H04-11912-M1 Unknown
34 H04-11912-M2 Unknown
35 H04-11912-M3 Unknown
36 H04-11912-M4 Unknown
37 H04-11912-M5 Unknown
38 H04-11912-M6 Unknown
39 H04-11912-M7 Unknown
40 H04-11912-M8 Unknown
Rotor-Gene Q Series Software 2.3.1
VIRTUAL MODE
