# Supplementary material for: Optimizing an ethanol-based fixative for enhanced nucleic acid preservation in cervical samples using a central composite design approach
Source: PLoS One. 2026 Jun 26;21(6):e0349088. doi: 10.1371/journal.pone.0349088 (PMC13308814; doi:10.1371/journal.pone.0349088)
Supplement: S3 File — (PDF) [file pone.0349088.s007.pdf]

Preliminary morphology characterization

1. Purpose of This Supplementary Analysis

This supplementary file presents the preliminary morphology assessment performed prior to the main comparative experiment. These early observations were conducted to evaluate the general performance of the novel fixative and gain an initial perception of cytomorphological preservation relative to the standard PreservCyt® solution.

Although informative, these data were not included in the manuscript main body to avoid confusing readers regarding samples reported in the main manuscript.

2. Sample preparation

Three cervical exfoliated cells were collected from consenting participants on 03 January and 06 January 2022 by Nilou Laboratory in accordance with the Declaration of Helsinki. Upon collection, samples were divided in two portions, and preserved in either the optimized ethanol-based fixative or the commercial PreservCyt solution. All samples were processed after 7 days of storage at room temperature.

3.Methods

Morphological preservation analysis

Fixed cervical exfoliated cells were collected from patients in either the optimized ethanol-based fixative or the commercial PreservCyt solution and were analyzed after seven days of storage at room temperature. Slides were prepared using the ThinPrep 2000 Processor (Hologic) and stained with Papanicolaou (Pap) stain. The slides were evaluated by a pathologist blinded to the preservation method.

4.Results

Comparison of morphological preservation

Cytological analysis showed that cervical exfoliated cells preserved in the final novel fixative exhibited excellent morphological integrity. Pap-stained slides revealed well-preserved cellular structures with no significant differences compared to samples stored in the PreservCyt. Both fixatives allowed for clear visualization of cellular features critical for diagnostic evaluation.

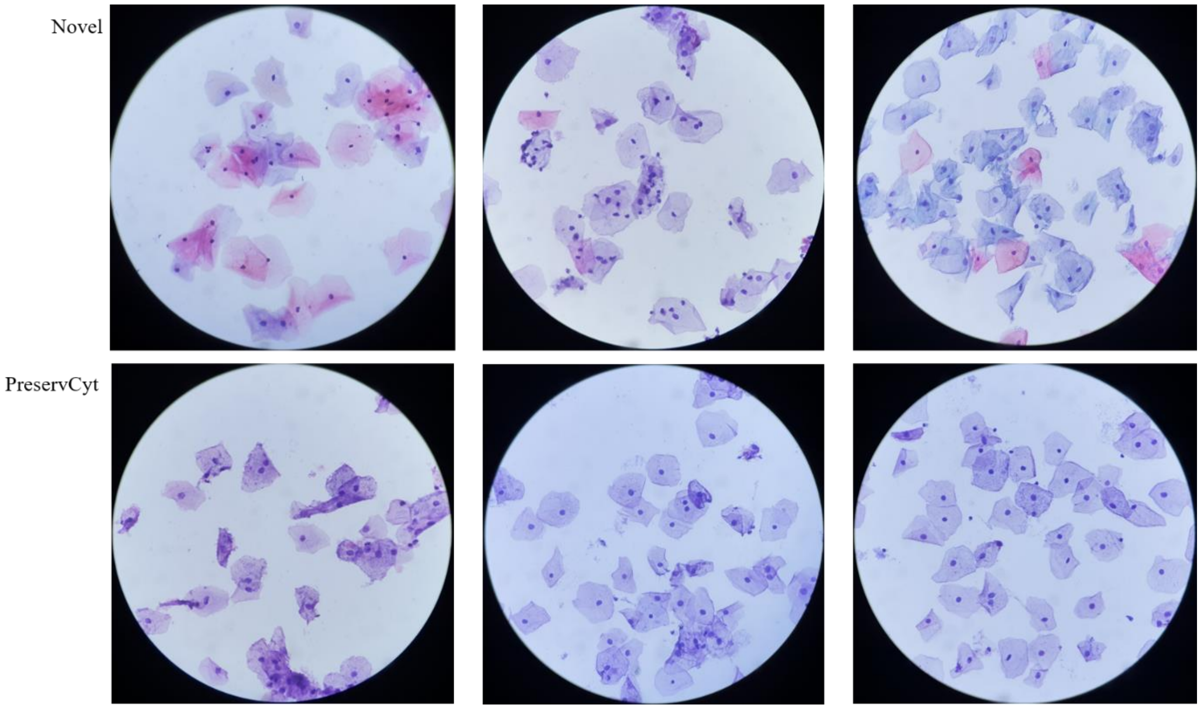

Fig. Clinical samples

Samples from three patients, tested in duplicate; preserved cervical swab specimens in both the novel solution and PreservCyt were satisfactory for cytopathology analysis.
